# A new bovine tuberculosis model for England and Wales (BoTMEW) to simulate epidemiology, surveillance and control

**DOI:** 10.1186/s12917-018-1595-9

**Published:** 2018-09-04

**Authors:** Colin P. D. Birch, Ashley Goddard, Oliver Tearne

**Affiliations:** Animal and Plant Health Agency, New Haw, Addlestone, Surrey, KT15 3NB UK

**Keywords:** Bovine tuberculosis, Stochastic simulation model, Wildlife reservoir, Cattle movements, Surveillance streams, *Mycobacterium bovis*

## Abstract

**Background:**

Bovine tuberculosis (bTB) is a zoonotic disease of cattle caused by *Mycobacterium bovis*, widespread in England and Wales. It has high incidence towards the South West of England and Wales, with much lower incidence to the East and North. A stochastic simulation model was developed to simulate *M. bovis* transmission among cattle, transfer by cattle movements and transmission from environmental reservoirs (often wildlife and especially badgers). It distinguishes five surveillance streams, including herd tests, pre-movement testing and slaughter surveillance. The model thereby simulates interventions in bTB surveillance and control, and generates outputs directly comparable to detailed disease records. An anonymized version of the executable model with its input data has been released. The model was fitted to cattle bTB records for 2008–2010 in a cross-sectional comparison, and its projection was compared with records from 2010 to 2016 for validation.

**Results:**

The fitted model explained over 99% of the variation among numbers of breakdowns in four defined regions and surveillance streams in 2010. It classified 7800 (95% confidence interval c. 5500 – 14,000) holdings within high incidence regions as exposed to infectious environmental reservoirs, out of over 31,000 cattle holdings identified as potentially exposed to such sources. The model was consistent with previous estimates of low *M. bovis* transmission rate among cattle, but cattle to cattle transmission was clearly required to generate the number of cattle cases observed. When projected to 2016, the model as fitted to 2010 continued to match the distribution of bTB among counties, although it was notable that the actual distribution of bTB in 2010 was itself a close match for its distribution in 2016.

**Conclusions:**

The close model fit demonstrated that cattle movements could generate breakdowns as observed in low incidence regions, if persistent environmental reservoirs such as wildlife maintained infection levels in the high incidence regions. The model suggests that environmental reservoirs may be a challenge for control, because, although many holdings are exposed to infection from wildlife or the environment, they are a minority of holdings. Large impacts on disease in wildlife will be required to avoid each individual transmission event to cattle.

**Electronic supplementary material:**

The online version of this article (10.1186/s12917-018-1595-9) contains supplementary material, which is available to authorized users.

## Background

Bovine Tuberculosis (bTB) is a worldwide bacterial disease of cattle with zoonotic potential caused by *Mycobacterium bovis*, which has increased in Great Britain from 500 to 700 cattle slaughtered for bTB control per year in the 1980s to 39,361 in 2016 [1, 2]. Currently human *M. bovis* infection is not a major problem in England and Wales, but the disease cost the English and Welsh governments £0.5 billion in the decade up to 2013 [3]. Scotland achieved Officially Tuberculosis Free Status (OTF) in September 2009, in recognition of the low incidence of bTB of native origin found in Scottish herds [4]. England and Wales also aspire to achieve OTF status before 2040, through deployment of an increased package of interventions to control all routes of transmission of disease [5, 6]. The aim is to substantially reduce bTB incidence as measured by the frequency at which herds experience ‘breakdowns’, in which restrictions and intense surveillance are applied following detection of bTB in one or more cattle.

Badgers were identified as a possible wildlife reservoir of infection for cattle in the early 1970s and Krebs et al. [7] concluded that there was strong evidence that badgers were a significant source of infection in areas with high bTB incidence [3, 8]. Despite the Randomized Badger Culling Trial (RBCT) of 1998–2007 confirming that badgers play a role in cattle bTB other questions have remained controversial [1]:

1. Can bTB be controlled without measures targeting badgers?
2. Can measures targeting badgers achieve net cost benefit?
3. Should culling or vaccination or both be used if badgers are targeted?

Bourne et al. [1] concluded that the answers to questions 1 and 2 were yes and no respectively. However others challenged these conclusions [9], and bTB has maintained or increased its range and incidence since 2007, while the net benefits of 5 years of proactive badger culling persisted at least 2.5 years beyond the end of the RBCT [10]. Moreover, various modelling and analytic methods have concluded that badgers or environmental sources contribute to a large proportion of herd breakdowns [11–13]. Hence culling and vaccination of badgers are potentially important tools for effective bTB control [5, 6].

The current escalation of control measures against bTB in Great Britain and the availability of multiple options confound evaluation of individual control measures. A mechanistic simulation of bTB and its management allows comparison of the progress of the epidemic with and without individual control measures [11]. However, the most prominently published model of bTB incidence among cattle herds does not explicitly represent a wildlife reservoir of infection but an abstracted environmental pool of infection, which is maintained by the presence of infectious cattle [11]. Implicitly it assumes that infection in wildlife is not self-sustaining, which is an issue of uncertainty [3]. Many hold the view that bTB in badgers is self-sustaining in parts of the UK and that transmission from wildlife to cattle must be prevented to achieve bTB eradication in England and Wales [14, 15].

In addition to Scotland, there are strong contrasts in regional bTB incidence within the rest of Great Britain. Hence contiguous counties in North and East England with low bTB incidence are designated as the ‘Low Risk Area’ (LRA), in contrast to the ‘High Risk Area’ (HRA) in the South-west and a buffer zone in between (‘Edge’) [6]. Explaining the persistence of bTB in England and Wales as due to wildlife reservoirs may also provide a direct process maintaining the contrast between the HRA and the LRA.

The model described here applied the hypothesis that wildlife had an important role in maintaining bTB and the contrasts between regions of England and Wales. It aimed to reproduce the distribution of bTB observed in England and Wales using a combination of relatively static environmental disease reservoirs and transfer by cattle movements. The hypothesis can be tested within the LRA, because it implies that high incidence of bTB within the HRA is maintained by sources of infection that are absent from the LRA. The test of the hypothesis was whether transfer of disease by cattle movements to regions with low bTB incidence was sufficient to match observed incidence, if disease in regions with high incidence was substantially maintained by infection from local environmental reservoirs. The model explicitly simulated detection of disease in some detail, including distinguishing between 5 surveillance streams: regular and targeted herd tests, pre-movement tests, slaughter surveillance and post breakdown tests. This was both for simulation of surveillance options and to allow model outputs to be directly compared with field records to compare epidemiological pathways as well as geographic distribution.

## Results

**Fig. 1:**
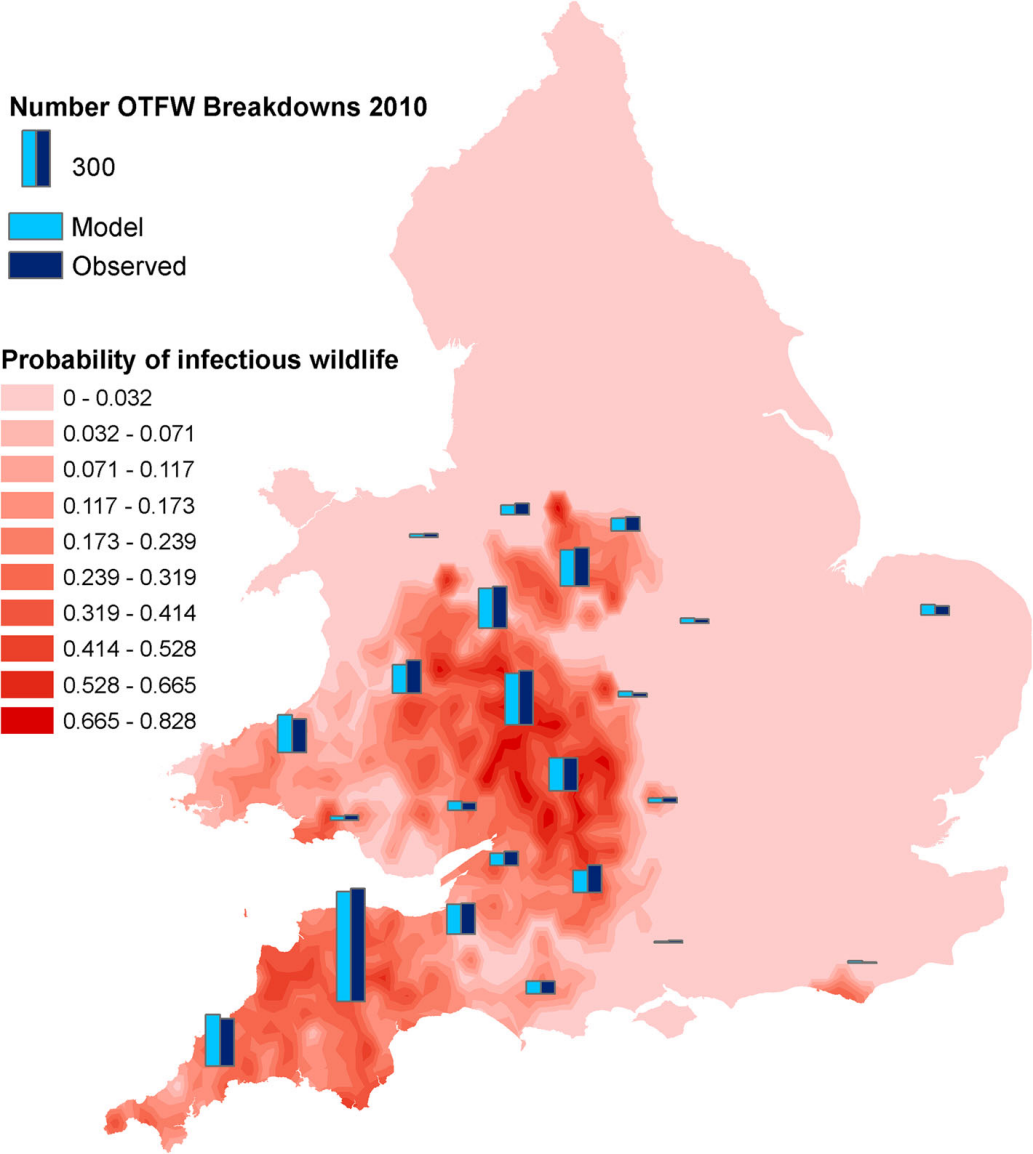
Distribution of infectious environmental reservoirs after fitting the model to bTB breakdowns in 2010. Background shades in red indicate the smoothed fitted local probabilities that environmental reservoirs were infectious. Vertical bars in light and dark blue indicate the numbers of modelled and observed breakdowns in counties or contiguous groups of counties. The pair of bars farthest East indicate numbers for the whole Low Risk Area

Model development estimated a set of parameter values that defined a baseline model, as explained in the Methods and the full model description (Additional file 1). The baseline model included a distribution of probabilities that holdings would include an infectious environmental reservoir for bTB, fitting it in the process of matching the model to the observed geographic distribution of breakdowns (Fig. 1) (Additional file 1, Part 2, Section 3 “Environmental infection state”). The proportion of environmental reservoirs that were infectious in the baseline model varied widely among geographic regions. Most of the infectious environmental reservoirs were within the HRA and high incidence areas of Wales, a few were within the Edge region and none were within the LRA, as defined by the model hypothesis that bTB in the LRA was due to transfer of disease by cattle movements.

**Fig. 2:**
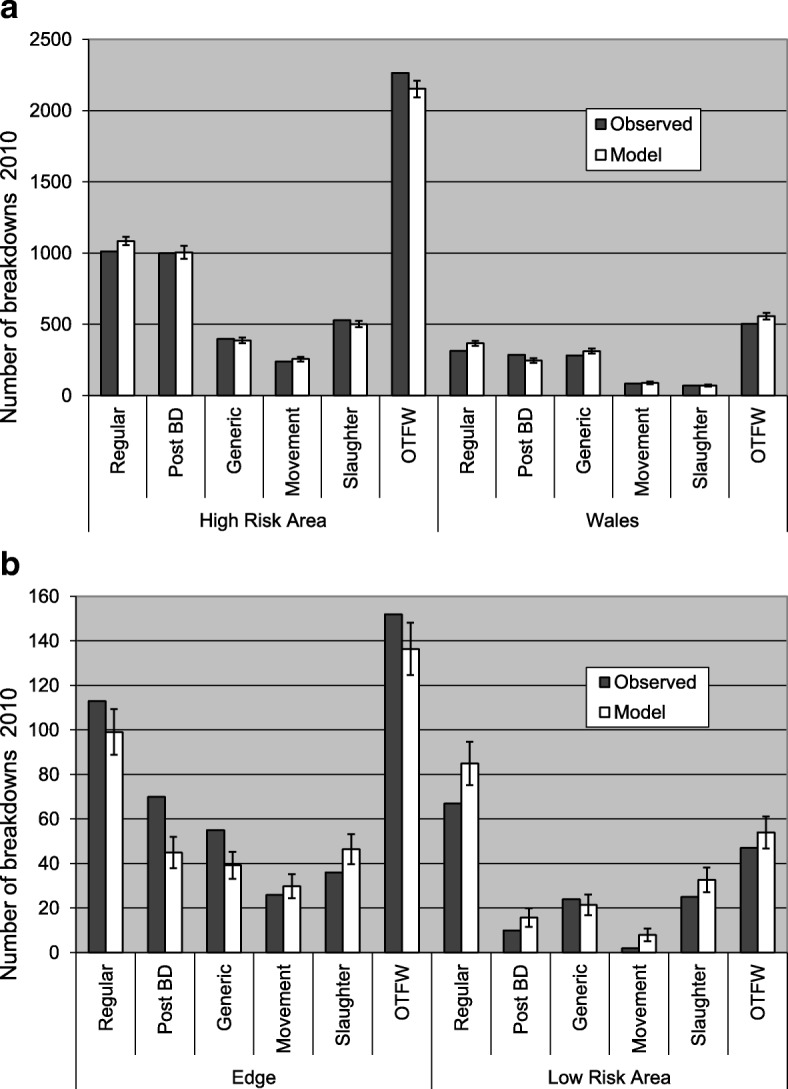
Comparison of model outputs with observed numbers of breakdowns. **a** The High Risk Area and Wales; **b** The Low Risk Area and the Edge region. Error bars indicate standard deviations among 10 simulations

In Fig. 2 the numbers of breakdowns generated by the model are compared with observed numbers in 2010. The model outputs match very closely the distribution of breakdowns among the surveillance streams, along with the strong contrast between the HRA (over 2000 Officially Tuberculosis Free status withdrawn (OTFW) breakdowns) and the LRA (less than 60 OTFW breakdowns), with R^2^ = 0.9930. However, the average *χ*^2^_24_ measure of deviation (see Methods) was 101.0 (Standard deviation (SD) 22.4), whereas the 95% upper confidence limit for the difference between two *χ*^2^_24_statistics generated by random variation would be 51.5 (see Additional file 1, Part 2, Section 2.5), so the deviation between the baseline model fit and the observations was clearly significant, demonstrating that the model fit was not the closest possible and model assumptions and simplifications caused significant errors.

The use of a *χ*^2^_24_ statistic to measure the fit of the model implied an assumption that the numbers of breakdowns were Poisson distributed among simulations. Indeed most SD of the numbers of breakdowns were close to the square roots of their averages, as expected from the Poisson distribution . The exceptions were the breakdowns in regular herd tests in the HRA and Wales, whose SD were about 12.5% lower than expected from the Poisson model; and breakdowns in the HRA at post-breakdown tests (SD 41% higher than expected) and total OTFW breakdowns (SD 25% higher than expected).

On average the model generated 54 (95 percentile range = 41–67) OTFW breakdowns in the LRA in 2010, compared with 46 reported for that year [2]. Hence the observed number of breakdowns was within the range consistent with the model outputs. There was no evidence that any source of infection was required in the LRA beyond the transfer by livestock movements that was simulated in the model.

**Fig. 3:**
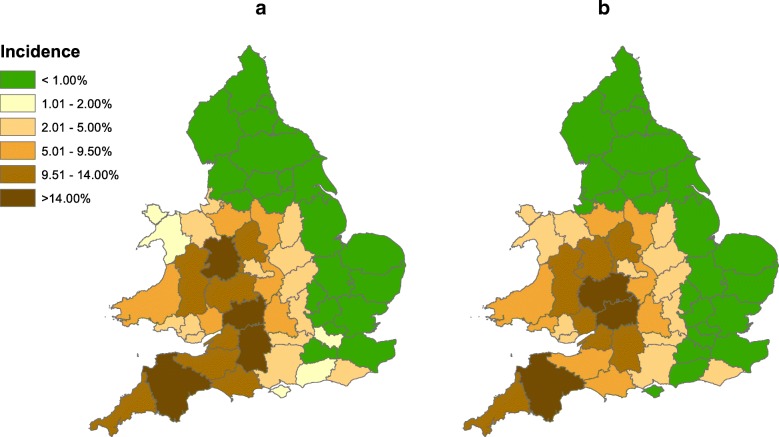
Annual incidence of herd breakdowns in England and Wales during 2014. **a** As observed; **b** As the average of ten outputs from the model. In both cases incidence is calculated for herds included in the model and breakdowns at those herds

**Fig. 4:**
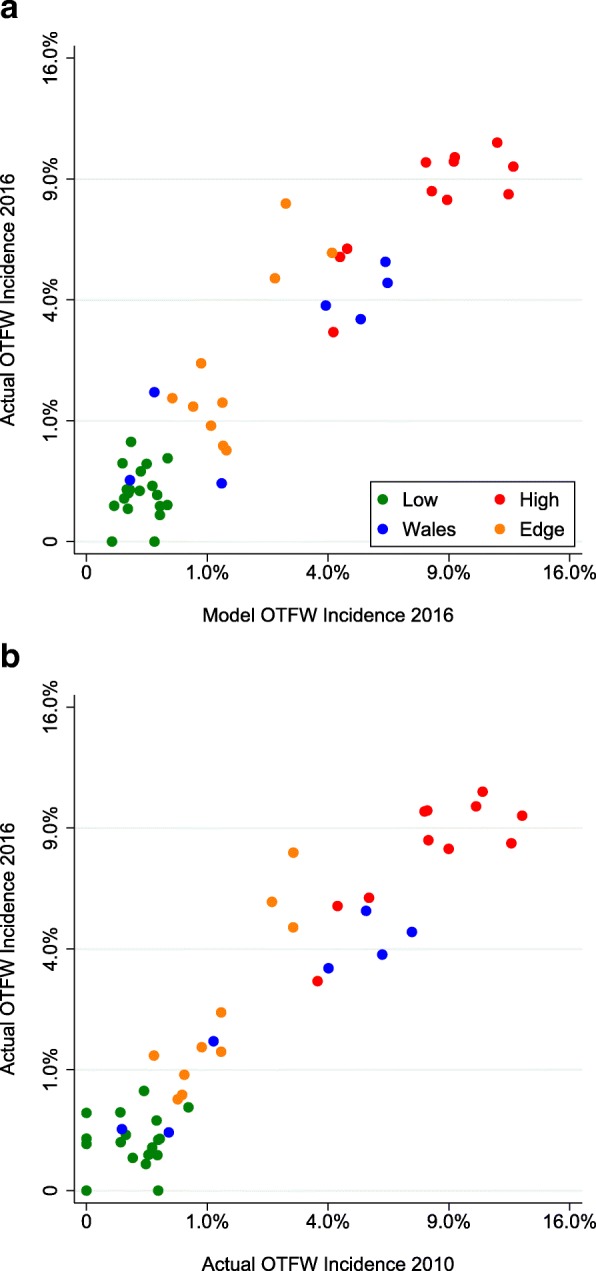
Relationship between the observed and modelled incidence of breakdowns by county in 2016. **a** The observed incidence compared with the model for 2016 (average of 10 outputs); **b** The observed incidence in 2016 compared with the observed incidence in 2010

**Fig. 5:**
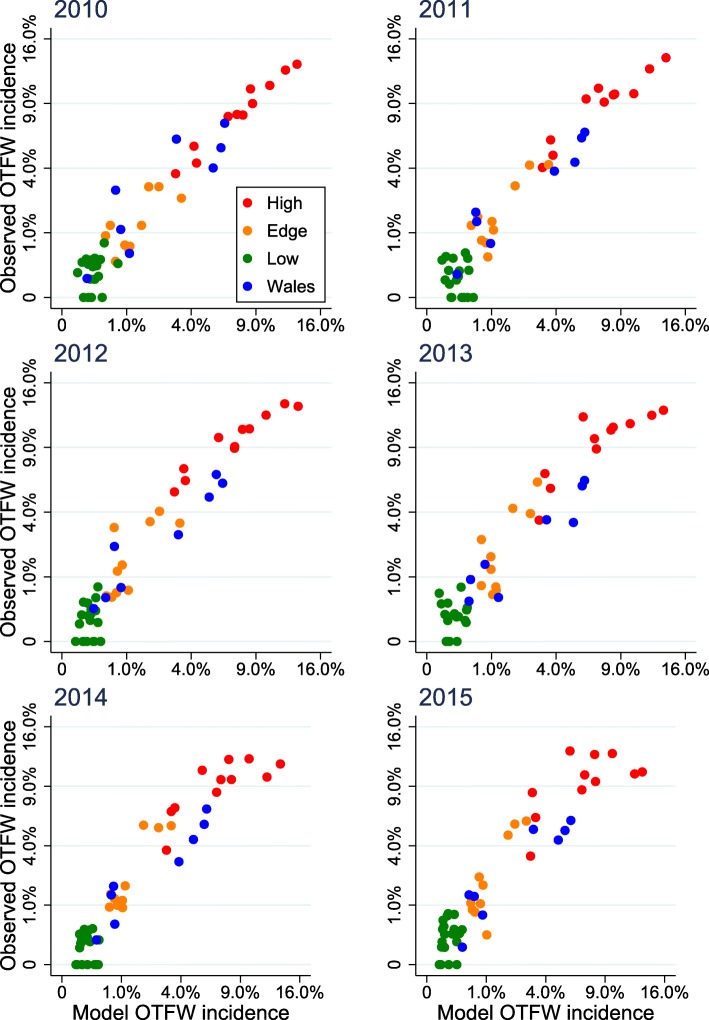
Relationship between the observed and modelled incidence of breakdowns by county during 2010–2015

The outcome of a validation test is displayed for 2014 in Fig. 3 as a map comparison based on the bTB surveillance report for 2014 [16]. The model outputs for 2014 match the major features of the observed distribution of bTB, particularly the strong contrast between high incidence within the HRA and parts of Wales and much lower incidence in the LRA. A plot comparing the model with observed breakdown incidence in individual counties in 2016 demonstrated that the relationship between model predictions and observed incidence remained consistently close across the full range from 0 to 12 breakdowns per 100 herds yr.^− 1^ (Fig. 4a), with R^2^ = 0.8866 (Data in Additional file 2). After square root transformation, differences between model predictions and observed incidences seemed to be more or less uniform relative to predicted values. A Cook-Weisberg test found no evidence of heteroscedasticity (*χ*^2^_1_ = 0.12, *p* > *χ*^2^_1_ = 0.72, Stata 15). However, the relationship between observed incidences in 2016 and 2010 was very similar to the relationship between observed incidence in 2016 and model predictions (compare Fig. 4b with a ), with R^2^ = 0.8901 (Data in Additional file 2). Implicitly the model achieved its match to 2016 mainly by generating little change from 2010. Comparison between observed bTB incidence and model outputs from 2010 to 2015 suggested a gradual deterioration in the match, although the distinctions between counties in the HRA, Edge and LRA regions and Wales were notably maintained (Fig. 5). The closer match in 2010 appeared to be mainly due to the close fitting of incidences in the HRA counties with highest incidence, as illustrated by Fig. 1; by 2016 the relative values among the counties with high incidences had shifted in ways that were not matched by the model.

The environmental reservoirs were particularly important for this model, being the source of about 36% (SD among years 2%) of individual cattle infections in unrestricted herds during 2000–2016, while cattle to cattle transmission was the source of the remaining 64% (Data in Additional file 3). Moreover, infections from environmental reservoirs often or usually took place in previously uninfected herds, while, by definition, cattle to cattle transmission only took place in herds that were already infected. Cattle to cattle transmission must be followed by movement of infected cattle between herds to introduce infection to new herds. However, among the 6.80 × 10^6^ cattle in the model, there were 1.95–2.04 × 10^6^ transfers to other herds per year during 2008–2010, and 1.61–1.76 × 10^6^ movements to slaughter (Additional file 4). Thus many or most infected cattle were detected before they left the herd where they were infected, while those that moved were almost as likely to move to slaughter as to transfer to another herd. Hence introduction of infection to previously uninfected herds was mainly due to transmission from environmental reservoirs.

**Fig. 6:**
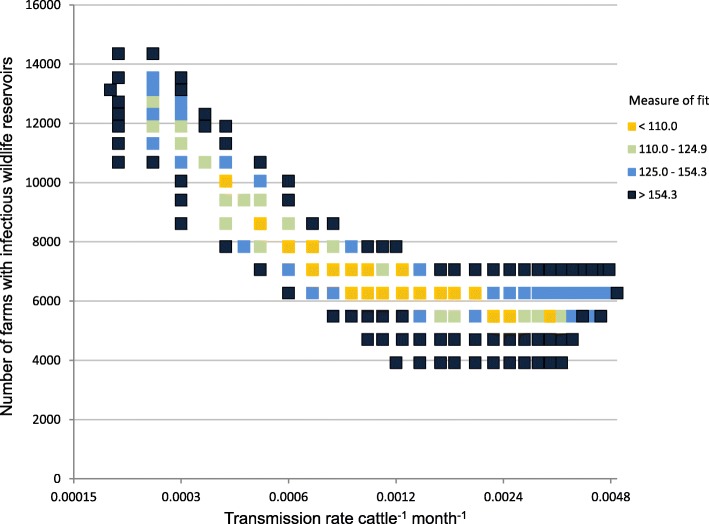
Fitting parameters of transmission of *M. bovis* from environmental sources. The figure shows the dependence of model fit on the number of holdings exposed to *M. bovis* in environmental reservoirs and the rate of transmission of *M. bovis* from an environmental reservoir to local cattle. All other parameters were kept at their baseline values. Low values of the measure of fit indicate combinations of the parameter values at which the model matches observations relatively closely. Measures of fit are based on 10 simulations per point. The horizontal, transmission rate axis is plotted on a log scale but labelled with back transformed values

The two parameters determining the environmental contribution, the number of infectious environmental reservoirs *N*_*W*_ (restricted to the HRA, Edge and Wales regions) and the environmental transmission parameter *h*, were varied, while keeping all other parameters at their baseline values, to find the potential range within which they could be consistent with observed bTB and its distribution among surveillance streams. Outputs from this sensitivity analysis suggested that the number of infectious environmental reservoirs was more constrained than the transmission rate (Fig. 6). It may be noted that the number of infectious environmental reservoirs is presented on a linear scale while the transmission rate is log-transformed. More infectious environmental reservoirs were required at lower transmission rates, but, even taking account of this trade off, the bulk of infection from environmental reservoirs should be taking place at only 5500–14,000 holdings. Note that these would be holdings with *possible* transmission of *M. bovis* from the environmental reservoir to cattle: infection of cattle from the environmental reservoir may be infrequent at many of these holdings.

## Discussion

One of the benefits of a mechanistic model is that it provides a quantitative description of the system it models that follows current understanding, especially if its outputs are a reasonable fit to observations. Quantification builds and challenges understanding, even though the numbers are approximate and uncertain. The current model’s description of the bTB epidemic in England and Wales is that introduction of *M. bovis* to previously uninfected herds is mainly driven by environmental reservoirs in areas of endemic infection, probably within a range from 5500 holdings (*N*_*W*_ in Table 1) exposed to environmental sources that would infect up to 1 in 20 cattle year^− 1^ (*h* in Table 1) to 14,000 holdings exposed to infection of 1 in 350 cattle year^− 1^ (Fig. 6). Infected cattle also transmit infection to other cattle at a rate of about 0.11 new infections per infected animal month^− 1^ in a herd of 200, increasing with herd size (see Methods and Additional file 1). These conclusions can be reached because the number of cattle detected with disease is too large relative to the number of breakdowns to be generated just by infection from environmental reservoirs. On the other hand, the number of cattle detected with disease is too small to generate the number of breakdowns observed just by cattle movements and by infection that persists in cattle at the end of breakdowns. Moreover, the persistent contrast in bTB incidence between the HRA and LRA, particularly among recurrent breakdowns, strongly suggests a link with location, such as localised environmental reservoirs.

**Table 1:** Main epidemiological parameters with baseline estimates and ranges

Parameter definition	Symbol	Estimate (Range)	Source / Derivation
Initial number of undetected infected herds	*Z*	4400 (1850–5050)	Fitted so that simulation matched the total number of breakdowns in 2008 within 100, i.e. between 4849 and 5049.
Number of holdings in England and Wales with infectious environmental reservoirs.	*N*_*W*_	7840 (SD ± 73) (5500–14,000)	Determined by the regional probabilities that individual holdings will have infectious environmental reservoirs (*P*_*W*_).
Probability that individual holdings have infectious environmental reservoirs, dependent on a classification based on county and TB history.	*P*_*W*_	0–0.828	The probabilities are fitted to local breakdown frequencies, see Fig. 1 and Methods.
Environment to cattle transmission rate per head of cattle at holdings with an infectious environmental reservoir.	*h*	0.0006 (0.0004–0.004) mth^− 1^	See Fig. 6.
Cattle to cattle transmission rate (per month) per infected animal in a herd of 200 cattle.	*β*	0.113 (0.10–0.14) mth^− 1^	Fitted to observed numbers of reactor animals at disclosing tests in HRA and LRA. Close to a previously published estimate [22].
Power law determining degree of density dependence of cattle-cattle transmission. (0 matches density dependence, while a value of 1 matches frequency dependence.)	*q*	0.5 (0.3–1.0)	Partial density dependence was demonstrated previously [22], but model outputs had low sensitivity to this parameter’s precise value.
Probability of a persistent infection at the end of a breakdown in herds > 300 cattle, given that infected cattle remained after the disclosing test or there was at least 1 additional reactor.	*c*_*p*_	0.6 (0.3–0.9)	Fitted to the observed number of breakdowns detected by post breakdown tests, especially in the Edge and Low Risk regions. Probability was lower in smaller herds, see Additional file 1.

‘Symbol’ is the symbol used in formulae. ‘Estimate (Range)’ indicates the baseline value, with a range indicating the potential uncertainty of the estimate, or a range dependent on location. Parameter values outside the stated ranges are likely to be associated with substantially worse model fit, or an infringement of reasonable constraints

The model developed here achieved a reasonable fit to the distribution of bTB among surveillance streams and regions and among counties, which was robust up to at least 6 years, (Figs. 2, 3, 4, 5). Therefore the distribution of bTB in England and Wales can be described based on a hypothesis that there is a relatively static reservoir of infection around some herds in the relatively high incidence regions, combined with transfer of infection by cattle movements. Model deviations from observed distributions of breakdowns were significant, but those deviations could be due to the technical limitations of the model, including assumptions that parameters have been constant across large areas and several years. The model fit to observed disease in 2010 (Figs. 1, 2) was part of the process of model development, not a demonstration that the model was valid. As described in the Methods below and full model description (Additional file 1), the model includes many parameters, which can be indefinitely extended through regional and temporal variation, so the baseline set of parameter values can be assumed to be just one of an indefinite number of alternative sets. The model structure and parameterisation could also benefit from further investigation and development.

A previous bTB model estimated just seven parameters using Approximate Bayesian Computation (ABC) with sequential Monte Carlo sampling, but required significant simplification, including no regionalisation and no differentiation between surveillance streams [11]. This previous model also identified an environmental reservoir of infection as important, but assumed that it was not self-sustaining but accumulated in the presence of infected cattle. Mechanistically this is very different from the reservoirs of infection in this model, which are assumed to be self-sustaining, although reservoirs of infection can become extinct, a feature of the model that may be used more in future applications. However in practice the two models converge, because they both simulate geographic regions in which high incidence of bTB in cattle is associated with substantial local reservoirs of infection at a minority of holdings.

Another difference between the model developed here and that of Brooks-Pollock et al. [11], which generates more differences between their predictions, is that, whereas most infected cattle are detected within this model and it has no latent period, the previous model assumed a long latent period lasting years with low detectability in cattle, which resulted in most infected cattle being slaughtered undetected [11]. However, most models of tuberculosis in cattle use relatively short periods of 1–8 weeks in which detection of infection is reduced, similar in duration to the assumption of this model [17]. The assumption of much longer periods with low detectability in cattle is contradicted by experimental evidence [18–20], although it is difficult to be certain about which features of experimental infection match natural infection. Moreover, assuming a high proportion of cattle are detected can generate distributions of breakdowns between the HRA and LRA and among counties that match observations (Figs. 2, 3, 4, 5), whereas the previous model overestimated numbers of breakdowns in counties with low bTB incidence and generated increasing numbers of breakdowns in the LRA instead of declining numbers (compare extended data Fig. 3c in [11] with Fig. 4 here, noting that numbers of breakdowns should have matched more closely than incidences), although the previous model used a different metric for fitting, which could also have contributed to this difference. The differences between the models will lead to them generating different predictions about the impact of interventions. For example, the previous model predicted that whole herd culls would reduce the number of herd breakdowns more than other interventions, whereas the current model would predict that whole herd culls would have relatively little impact.

In the current model many holdings are exposed to infectious environmental reservoirs, which are a source for many infections, but, except for a few small areas, holdings with infectious environmental reservoirs are a minority, even within regions with a high incidence of bTB (Fig. 1). According to the model fit, environment to cattle infection mainly takes place at less than 14,000 holdings, while the model set up identified over 31,000 holdings that might include infectious environmental reservoirs at the start of 2008 (Additional file 1: Table S4). Even at those holdings with infectious environmental reservoirs in the model, few or no infections from the environment to cattle take place each year. Hence regional control measures aiming to reduce infection from the environment may require substantial inputs to achieve impacts. For example, many wild animals must be culled or vaccinated for each transmission event to cattle that is avoided. Therefore targeting of control measures using statistical models, or models like this one could be beneficial. Some control measures, such as biosecurity of buildings, may also be more suitable for targeting, especially given recent evidence that interactions between badgers and cattle are rare and mainly take place at specific, identifiable locations [21]. However, improved biosecurity might reduce the cost-effectiveness of eradicating bTB from wildlife.

The benefit from quantitative, mechanistic models such as this, in comparison with broader scientific reasoning, is that quantifying impacts can have an important influence on conclusions. It was perhaps disappointing that the model’s match to the observed distribution and dynamics of bTB was achieved by predicting little change, but the insight is valid and potentially useful. This model is not inert and it can generate changes in response to substantial measures such as widespread vaccination. Confidence that badgers are a source of *M. bovis* infection in cattle might suggest that reducing bTB among badgers is a way to reduce bTB incidence in cattle [9]; however, the insight that badger to cattle transmission may be unusual although very important suggests that controlling bTB in badgers may be a challenging option, even if it is essential.

## Conclusions

The BoTMEW model achieved a reasonable fit to the observed numbers and distribution of BTB breakdowns in 2010 and maintained a good match to the relatively stable state of bTB since then. It demonstrated that transfer by cattle movements was sufficient to generate observed levels of bTB in the LRA, if higher incidence in the HRA was largely maintained by infection from environmental sources. To match features of bTB in England and Wales, such as the high observed numbers of breakdowns detected by post-breakdown surveillance, the model fit suggested that only a minority of herds in the HRA are exposed to environmental sources of infection. Therefore cost-effective control of bTB by reducing *M. bovis* transmission from the environment may be challenging. However, the outputs of any model depend on its structure and parameterisation, and, since the BoTMEW model has not yet been run with a wide variety of fitted parameter sets, its behaviour has not yet been fully investigated, and comparison with other models would be advisable.

## Methods

**Fig. 7:**
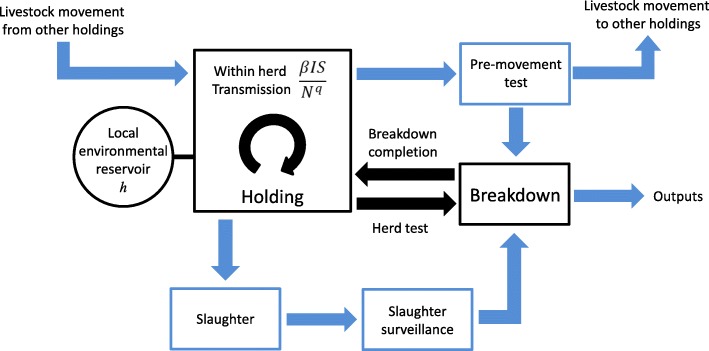
Flow diagram summarising surveillance and disease transmission in the model. The relationship between the transfer and detection of TB through daily livestock movements (blue arrows) and epidemiology and detection resolved monthly at holding level (black). The pre-movement test and slaughter surveillance streams are distinguished from herd tests. Symbols in the transmission equations are: *N* = number of cattle in herd, *I* = number of infected cattle, *S* = number of susceptible cattle, *β* = cattle to cattle transmission parameter, *q* = power law for cattle to cattle transmission and *h* = environment to cattle transmission parameter

The Bovine Tuberculosis Model for England and Wales (BoTMEW) is a spatially explicit, stochastic, dynamic simulation model of *M. bovis* transmission and control throughout England and Wales at the resolution of farm holdings and individual infected cattle. It focuses on the spread of *M. bovis* to individual herds by import of infected cattle, or by transmission from a local environmental reservoir (Table 1), and on the detection of infected herds by surveillance (Fig. 7, Table 2), which mainly relies on the single intradermal comparative cervical tuberculin (SICCT) skin test and detection at slaughter. The model also represents transmission among cattle within herds and the restrictions on herds detected as infected (‘breakdowns’), while they are being cleared of infection. However, these within herd processes are simplified compared with specifically within herd models [17, 22]. The model is fully described and explained in a model description (Additional file 1). Here the main features of the model are described, focussing on transmission by cattle movements and from environmental reservoirs. An anonymized executable of the model with a full set of inputs has been released [23].

**Table 2:** Main surveillance parameters with current estimates

Parameter definition	Symbol	Estimate (Range)	S-T range	Source / Derivation
Maximum probability of detecting one infected animal in a herd test using the SICCT skin test	*a*	0.48 (0.36–0.63)	0.45–0.53	In literature [22, 33] and estimated by fitting. The number of breakdowns disclosed by slaughterhouse surveillance is sensitive to this parameter.
Maximum probability of detecting one infected animal when it is moved from a holding within an area where pre-movement testing is applied.	*a*_*m*_	0.32 (0.24–0.42)	N	Detection reduced by exemptions from pre-movement testing. The recorded number of pre-movement skin tests was about ½ the number of movements from areas with pre-movement testing. Detection > 0.48/2 because infected cattle were more likely to be in movements without exemptions.
Infection duration until SICCT reaches maximum sensitivity	*t*_*L*_	35 (20–200) d	N	Experimental evidence [20].
Specificity of an individual SICCT skin test.	*sp*_*Sk*_	0.9998	N	The estimate of Goodchild et al. [34].
Proportion of age class *i* that is tested in a regular herd test	*w*_1_ *w*_2_..*w*_5_	0 0.5–1.0	0 0.50–0.78	Fitted to observed numbers of regular herd tests.
Frequency of generic herd tests on infected herds	*g*_*i*_	Location dependent	0.020–0.09 mth^−1^	Fitted to observed numbers of breakdowns detected by generic herd tests.
Frequency of generic herd tests on uninfected herds	*g*_*u*_	Location dependent	0.0012–0.04 mth^−1^	Fitted to observed numbers of generic herd tests.
Maximum probability of detecting an infected animal at slaughterhouse surveillance.	*a*_*s*_	0.875 (0.70–1.00)	0.557–0.875	Estimate may be high because of factors and practices absent from the model, including approved finishing units and age effects [35, 36]. Sensitivity increased during 2008–2010.
Maximum probability of confirming an infected reactor.	*a*_*c*_	0.40	N	Fitted to number of OTFW (confirmed) breakdowns. This value underestimates the number of OTFW animals. (OTFW animals are unevenly distributed among breakdowns.)
Time required for lesions to reach maximum detectability.	*t*_*Ls*_	35 d (20–200) d	N	This and other models seem to fit using a wide range of values for related parameters [22]. Reports that a high proportion of young reactors have visible lesions indicate a low value [36].

See legend for Table 1. ‘S-T range’, i.e. ‘Spatial-temporal range’ indicates the actual range of values for the parameter used for the baseline fitted model in England and Wales during 2008–2010. ‘N’ indicates that only a single value was used for the parameter

### Introduction of *M. bovis* by cattle movements

Movement is the only process in the model by which cattle can transmit *M. bovis* to another herd. Direct transmission of *M. bovis* between cattle ‘over the fence’ is not explicitly simulated, because *M. bovis* infection rates are low and cattle to cattle contacts within herds are much more frequent than between neighbouring herds. There is no clear evidence of cattle to cattle transmission between contiguous herds that have not exchanged any animals or shared pasture [3]. In any case, actual transmission ‘over the fence’ is difficult to distinguish from successive infections from a shared environmental source.

Cattle movements were simulated by replaying the movements recorded in the Cattle Tracing Scheme (CTS) for England and Wales every day during the three calendar years 2008–2010. The CTS data had already been georeferenced and paired by the Data Systems Group at APHA to allow analysis of movement patterns, and series of movements via markets and other intermediate locations had been simplified to transfers between herds, or movements from herds to slaughter [24]. The quality of CTS data is more than adequate for this application: for example, missing movements or movements with errors will mostly result in incomplete pairs of movements, which constituted only 1.4% of all paired movements during 2008–2017 (Additional file 4). Further movements were omitted because CTS data excluded movements between pairs of holdings that arranged exemptions from reporting called ‘CTS links’, which involved about 35% of cattle holdings in 2008 but only included a small proportion of all movements [25]. CTS links no longer exist after being phased out from July 2016, a change that has not had an obvious impact on the total number of movements recorded (e.g. see Additional file 4).

The network of source and destination herds was also based on CTS data, but was static for each simulation, i.e. the list of herds and their cattle populations did not change during a simulation. The baseline set up included 6,802,196 cattle in 73,371 herds. Because the resolution of CTS is at holding level, the model treats each holding as consisting of a single cattle herd and its associated environmental reservoir. The restriction of holdings to a fixed list with only one herd at each holding meant that reference numbers of observed breakdowns for this study could be slightly fewer than in official statistics.

If a movement took place from an unrestricted herd including at least one infected animal, and the age class of an animal moved matched that of an infected animal, the infected animal had a chance of being selected for the movement. The cattle at each herd were allocated to five age classes: 0–42 days, 43 days to 15 months, > 15–30 months, > 30–60 months and > 60 months. By regulation calves up to 42 days old are too young for SICCT testing, while 30 months used to be the maximum age in the UK of cattle that could enter the human food chain due to bovine spongiform encephalopathy; the division at 15 months divides cattle up to 30 months roughly in half in time and numbers, while the division at 60 months divides the number of cattle older than 30 months roughly in half. Selection was stochastic, based on an assumption that all animals within an age class in a herd had equal probability of being selected. If the movement was to another herd, a pre-movement test might be applied to the moving animals, depending on the local control regime. If there was no reactor to tests, information about the infected animal was moved to the destination herd. If the movement was to slaughter, surveillance at slaughter was checked for every infected animal. If any were detected a breakdown was started at the source herd. Any infected animals that were not detected at slaughter were recorded as an output from the model.

### Transmission of *M. bovis* from environmental reservoirs

Every holding was assumed to have a local environmental reservoir, treated as being independent from environmental reservoirs in other holdings (Fig. 7). In the simulations reported here, the reservoir was assumed to be either infectious or not infectious for the years 2008–2010 of each simulation, an assumption influenced by studies at Woodchester Park, UK showing that a wildlife reservoir can be very local and maintain a stable spatial distribution for several years [26]. However, although Woodchester Park has been carefully studied for many years, it may not be typical of badger populations within the HRA. The model simulates potential changes to the infection state of environmental reservoirs during 2011–2016 (see Additional file 1).

An iterative approach was used to classify holdings in England and Wales into one of nine classes for the probability that infectious environmental reservoirs were present at the start of a simulation (see Additional file 1). It was assumed that all holdings in the LRA had zero probability of having an infectious environmental reservoir, as well as all other holdings with no bTB history and in areas with local bTB incidence < 1 reactor / 1000 cattle / year, estimated within a grid of 6.25 km^2^ hexagonal cells [27].

For model fitting, all the probabilities that environmental reservoirs were infectious were increased or decreased by equal multiples, to preserve the relative distribution of infection in the environment, except the maximum probability was 1.00. Parameter settings for scenarios were summarized by reporting the number of infected environmental reservoirs, *N*_*W*_. Within holdings that had infectious environmental reservoirs, every cow had the same probability of becoming infected from the environment each month, which was set by the environment transmission parameter *h*. Hence the actual distribution of environmental infectiousness was approximated by environmental reservoirs only having two values of infectiousness: 0 or *h*.

The potential ranges of the average infection rate from the environment *h* and the number of infected environmental reservoirs *N*_*W*_ were investigated by varying these two parameters from their baseline settings. The relationship between these values and measures of model fit could potentially indicate limits to their ranges. Although environmental infectiousness and the number of infected environmental reservoirs could compensate for each other’s influence on total numbers of breakdowns, their relative values might be limited by the proportion of breakdowns that were disclosed by post breakdown tests, for example.

### Detection of *M. bovis* infection in unrestricted herds

The model was designed to generate outputs that could be directly compared with surveillance observations, which were carefully simulated as 5 distinct surveillance streams: regular and targeted herd tests, pre-movement tests, slaughter surveillance and post breakdown tests (Fig. 7). Regular herd tests are tests carried out to meet the minimum regionally required frequency of bTB testing (currently every 4 years in the LRA and annually in the rest of England and Wales), while targeted herd tests represent all surveillance not included in the other 4 streams. The model also distinguished cattle infections and hence breakdowns as confirmed by visible lesions or bacterial culture, which is the main reason for classifying breakdowns as OTFW, or unconfirmed. Further detail is provided in the full model description (see Additional file 1).

### Transmission within herds and breakdown processes

Cattle to cattle transmission was calculated once per month in herds with infected cattle, to compromise between execution speed and accuracy, while coordinating with the 2 month time intervals required for short interval testing within breakdown herds (Fig. 7). Previous models have been unable to decisively select between strongly contrasting assumptions about the development of individual infectiousness [22]. All models consider transitions between a susceptible state and an infectious state, but many models also include a latent period between infection and becoming infectious, or a period in which cattle are less reactive to surveillance tests, which may be before or after they become infectious [17, 28]. The direct evidence for a latent period and for a delay before infected cattle become reactive to surveillance tests is limited and possibly consistent with both periods being short [18], while parameters for different aspects of epidemiology may compensate for each other, e.g. a reduced transmission rate could compensate a shorter or omitted latent period [17]. Here each cow was assumed to have constant infectiousness *β* from the moment of infection, as assumed by the susceptible-occult-reactor (SOR) model of Conlan et al. 2012 [22]. A power law parameter, with a potential value between 0 and 1 was used to adjust the dependence on herd size of cattle-to-cattle transmission within a herd (Table 1) [22].

Several within-herd models of *M. bovis* transmission and control have been developed [17, 22, 28–30]. In contrast, the primary focus of this model was to simulate infection dynamics into and within unrestricted herds, to provide a framework for exploring the impact of control options on undetected infection. Nevertheless some representation of breakdown resolution was required, mainly because periods of restriction of herds at high risk of infection, and potential persistence of undetected infected cattle were essential to the dynamics of the system [22]. At the start of each breakdown, its duration, the numbers of confirmed, unconfirmed and false positive reactors, and whether an undetected infected cow would remain at the end of the breakdown were determined, based on the state of the holding affected (see Additional file 1). The overall dynamics of the model have low sensitivity to the duration of breakdowns, while the number of new reactors during a breakdown has no effect on any other process or output from the model.

### Model initialisation and setup

The model was seeded with infected herds taken from a chronological list of herds that had reported breakdowns starting from 1st January 2008 onwards. If necessary, the number of herds seeded was adjusted until the average number of simulated breakdowns in 2008 was within 100 of the observed number, i.e. within the range 4850–5050. By 2010, the year used for model fitting, almost all these herds had either lost their infection or been detected. Herds in breakdown at the start of the simulation, or that recently had breakdowns were also identified, to minimize initial changes in model behaviour (see Additional file 1 and the full model set up [23]).

### Programming and quality assurance and verification of code

The model was coded in Java Standard Edition version 7 using the Eclipse Integrated Development Environment (http://www.eclipse.org/) and the Git version control system (http://git-scm.com/). The code was written using test-driven development methods, which involve testing each model component in isolation to verify against the agreed model algorithms, as well as protecting against new components unexpectedly interacting with pre-existing code [31]. Distinct versions of the software were created as new features were added, with every code change logged and attributed to its author in the version control system.

The model is single threaded but multiple copies can be started from separate working directories on computers with multiple CPU cores. Logging and outputs data are saved as text files in CSV format in the working directory. Additional scripts were created to assist with setting up the multiple working directories for parallel runs, for scenario exploration, and to aggregate results data.

Third party open source libraries used in the model include the following.

- Mathematical functions: Colt high performance scientific computing library, developed at CERN (http://acs.lbl.gov/software/colt/), and the Apache Commons project (http://commons.apache.org/).
- Logging framework: SLF4J (http://www.slf4j.org/) and the Apache logging project (http://logging.apache.org/log4j/1.2/).
- Testing framework: JUnit (http://junit.org/) and Mockito (http://code.google.com/p/mockito/).

### Model outputs

All simulated herd breakdowns during a run were recorded, including start date and duration, the surveillance stream that disclosed the breakdown, and the numbers of reactors and confirmed reactors. At the end of a simulation, data was also output for current undetected infected cattle, as well as a list of holdings with infected environmental reservoirs. In addition, summary information was output each year of the simulation for numbers of breakdowns disclosed by each surveillance stream, numbers of confirmed breakdowns, numbers of cattle infected from environmental reservoirs and from other cattle while herds were unrestricted, and total numbers of reactors, confirmed reactors and false positive reactors, including those detected during herd breakdowns. This summary information was provided for four regions: Wales, the LRA of England and two regions defined by county boundaries to approximate the HRA of England and the Edge region of England. (The regions for summary outputs were intended to be readily modified, so they were defined by counties rather than lists of individual holdings.) The hypothesis that *M. bovis* could only be introduced to herds in the LRA through cattle movements was tested by comparing model outputs with the observed number of OTFW breakdowns, because the total number of breakdowns in the LRA would be substantially dependent on the specificity of surveillance tests.

### Model fitting

Data on bTB breakdowns for model development and fitting was taken from the APHA Vetnet database for 2002–2011, which has been superseded by the Sam database, APHA’s herd registration and notifiable animal disease control and surveillance system, which records, for example, details of herds, TB tests, TB incidents and the details of any slaughtered (reactors, slaughterhouse cases and direct contacts) and inconclusive reactor cattle. The model was fitted by cross-sectional comparison with data for 2010 (see Additional file 3). Due to technical constraints at the time of model development, this model could not be fitted using fully computerised algorithms such as ABC. Instead parameters were estimated using a combination of external parameter estimates, applying model constraints and through iteration, comparing among batches of simulations with ranges of parameter estimates informed by previous simulation outputs. However, model behaviour was carefully investigated and is reported in the full model description (Part 2, Section 4 Sensitivity Analysis in Additional file 1).

Formalised and computerised fitting algorithms such as ABC would have the advantage that they would identify posterior distributions for fitted parameter values that would allow estimation of the uncertainty of model outputs. However, when this model was developed in 2011, it was impossible to use methods such as ABC to fit a model as complicated as this. Even now, development of a model like this using ABC or other computer fitting algorithms is dominated by the challenges of parameter estimation, fundamentally affecting the model design and purpose. Fitting also requires decisions about model structure and the prior distributions of parameters that may be subject to biological dispute. Models with different purposes and priorities can be fitted different ways to complement each other’s strengths.

Each simulation included over 3000 breakdowns per year, so variation among simulations with the same set of parameter values was small relative to differences between simulations with different parameters. Hence 10 replicates were sufficient to evaluate each set of parameters. Because there was indefinite scope for improvement and there were alternative criteria for evaluating model fit, the set of parameter values used for sensitivity analysis and model exploration is referred to as the ‘baseline’ set of parameter values (Tables 1 and 2). For the baseline parameter set, 200 replicates were used to gain more precise estimates of its outputs and variance.

Fitting was most guided by a ${\upchi}_{24}^2$measure of deviation of the model from the actual number and distribution of breakdowns in 2010, which was calculated by comparing for each of the four summary regions the numbers of breakdowns from the five surveillance streams and the number of confirmed breakdowns, i.e. six times four = 24 comparisons. The ${\upchi}_{24}^2$measure was the sum of 24 terms of the form:

3 $$ {\chi}^2=\frac{{\left({N}_{\operatorname{mod}\mathrm{el}}-{N}_{\mathrm{observed}}\right)}^2}{N_{\operatorname{mod}\mathrm{el}}} $$

where *N*_model_ is the average number of breakdowns from model runs. This measure was expected to have an approximately *χ*^2^_24_ distribution, so it is referred to as the ‘*χ*^2^_24_ value’.

The *χ*^2^_24_ value was used to indicate potential ranges for individual parameter values by observing the impact on the average *χ*^2^_24_ value of varying parameters from their baseline values (Tables 1 and 2). The calculation of the *χ*^2^_24_ value can be seen implemented in a spreadsheet in Additional file 3. Note that this metric is the same for all the surveillance streams, although it will give higher weight per breakdown to deviations from streams detecting smaller numbers of cases. Based on the conservative argument of paragraph 2.5 of Part 2 of Additional file 1, an average *χ*^2^_24_ measure from 10 model replicates greater than 154.3 was treated as a significant deterioration of model fit at *p* < 0.05.

A *χ*^2^ metric was chosen for evaluating model fit to allow matching to the observed contrast between large numbers of breakdowns in the HRA and parts of Wales, relative to much smaller numbers in the Edge region and the LRA. Being count data, dividing squared differences by expected values was expected to correctly weight deviations from a wide range of observed values. A previous bTB model used squared deviations as its metric, which may have reflected its emphasis on the development of bTB over time [11].

As an approximation to a large dataset, the model was almost certain to deviate significantly from observed values. Therefore R^2^ was used as an index of the usefulness of the model, i.e. the portion of variation among data values after square root transformation that was explained by the model.

### Model validation

Fitting a model demonstrates that it can match a set of observations of the system it models, but it does not demonstrate how stable the model behaviour is outside the range it was fitted to. When a model has many parameters there is a risk of overfitting, which may lead to the model rapidly deviating from actuality outside the range of data it was fitted to. Now a few years have elapsed since the model fit to 2010, the baseline model was projected to generate the incidence of bTB among counties in England and Wales up to 2016 (% herds starting a new breakdown during the year) to compare with data on observed incidence, which is in the public domain [2] (see Additional file 2). As mentioned above and in the full model description (Additional file 1), potential dynamic changes to the infection state of environmental reservoirs were introduced and the cattle to cattle transmission rate was increased during 2011–2016. Projections into the future using the model can only use past movement data, so it was considered appropriate to use recycled movement data for this validation exercise. A previous study reported no clear trend in cattle movement patterns during 2002–2010 [32], and the original fitting period for the model, 2008–2010, was partly chosen to follow the impacts on cattle movements from introducing pre-movement testing during 2006–2007. A recent query of CTS data has confirmed that total numbers of cattle movements in Great Britain and seasonal patterns were stable during 2008–2017, with average annual paired non birth or death cattle movements = 6.68 × 10^6^ year^− 1^ during 2008–2010 and 6.65 × 10^6^ year^− 1^ during 2011–2016 (Additional file 4). Movements were generated for the projection by recycling the movements from 2008 to 2010, so movements in 2011–2016 repeated those recorded in 2008–2010 twice. To check that the model maintained a match to the observed distribution of bTB, modelled and observed incidences of bTB were compared visually and by regression among counties. Since the model was fitted to observed bTB in 2010, observed incidences in 2016 and 2010 were also compared with each other in an equivalent way.

### Anonymised model

For submission for publication, an alternative anonymised version of the model has been released [23]. The CPH identifiers have been altered to only retain the county location of each holding, and geographical locations have been removed. The summary outputs have been confirmed to be a close match at county resolution to the full model with the same parameter sets (Section 6, Part 2 of Additional file 1).

## Additional files

Additional file 1: Detailed functional description of a bovine tuberculosis model for England and Wales (BoTMEW), 41 pages. Part 1 Model specification. Part 2 Model setup and use. (DOCX 282 kb)

Additional file 2: A spreadsheet file with outputs from a recent run of the anonymised model with observed numbers of OTFW breakdowns in 2016 as displayed in Fig. 4. (ODS 1143 kb)

Additional file 3: A spreadsheet file with outputs from a recent run of the anonymised model with observed numbers of breakdowns in 2010 as displayed in Fig. 2. (ODS 115 kb)

Additional file 4: A spreadsheet providing annual numbers of movements recorded in CTS, to indicate the frequency of errors, numbers of transfers between herds during 2008–2010, and variation among years. (ODS 4 kb)

## Abbreviations

Baseline model: The model run using a set of parameter values, including variations of some parameters between regions and times, chosen because it achieved a good fit to observed records of bTB in 2010 and matched current knowledge of bTB epidemiology.
BoTMEW: Bovine tuberculosis model for England and Wales
bTB: Bovine tuberculosis
CPH: County parish holding identifier
CTS: Cattle tracing scheme
Edge: Buffer region between the HRA and LRA
*h*: *M. bovis* transmission rate from an infectious environmental reservoir per susceptible cow
HRA: High risk area
LRA: Low risk area
MS: Mean square error
*N*_*W*_: The total number of holdings with infectious environmental reservoirs present
OTFW: Officially tuberculosis free status withdrawn
P: Probability that a test statistic would be exceeded if a null model applied
R^2^: The proportion of variance explained by a regression model
RBCT: Randomized Badger Culling Trial
SD: Standard deviation
SE: Standard error
*χ*^2^: Measure of deviation with Chi-square distribution

## References

[1] Bourne FJ, Donnelly CA, Cox DR, Gettinby G, McInerney JP, Morrison WI (2007). Bovine TB: the scientific evidence. A Science Base for a sustainable policy to control TB in cattle. An epidemiological investigation into bovine tuberculosis.

[2] Quarterly publication of National Statistics on the incidence and prevalence of tuberculosis (2017). (TB) in cattle in Great Britain.

[3] Godfray HCJ, Donnelly CA, Kao RR, Macdonald DW, McDonald RA, Petrokofsky G (2013). A restatement of the natural science evidence base relevant to the control of bovine tuberculosis in Great Britain. Proc R Soc B.

[4] Bessell PR, Orton R, O'Hare A, Mellor DJ, Logue D, Kao RR (2013). Developing a framework for risk-based surveillance of tuberculosis in cattle: a case study of its application in Scotland. Epidemiol Infect.

[5] Wales TB (2017). Eradication Programme delivery plan. Welsh government.

[6] The Strategy for achieving Officially Bovine Tuberculosis Free status for England (2014). Department for environment, food and rural affairs.

[7] Krebs J, Anderson R, Clutton-Brock T, Morrison I, Young D, Donnelly C (1997). Bovine tuberculosis in cattle and badgers.

[8] Goodchild AV, Clifton-Hadley RS (2001). Cattle-to-cattle transmission of Mycobacterium bovis. Tuberculosis.

[9] King D (2007). Bovine tuberculosis in cattle and badgers. Department for Environment, Food and Rural Affairs.

[10] Jenkins HE, Woodroffe R, Donnelly CA (2010). The duration of the effects of repeated widespread badger culling on cattle tuberculosis following the cessation of culling. PLoS One.

[11] Brooks-Pollock E, Roberts GO (2014). Keeling MJ. A dynamic model of bovine tuberculosis spread and control in Great Britain. Nature.

[12] Donnelly CA, Nouvellet P. The contribution of badgers to confirmed tuberculosis in cattle in high-incidence areas in England. PLOS Currents Outbreaks. 2013;1 10.1371/currents.outbreaks.097a904d3f3619db2fe78d24bc776098 PMC3992815 24761309

[13] Green DM, Kiss IZ, Mitchell AP, Kao RR (2008). Estimates for local and movement-based transmission of bovine tuberculosis in British cattle. Proc R Soc B.

[14] Fitzgerald SD, Kaneene JB (2013). Wildlife reservoirs of bovine tuberculosis worldwide: hosts, pathology, surveillance, and control. Vet Pathol.

[15] Palmer MV (2013). Mycobacterium bovis: characteristics of wildlife reservoir hosts. Transbound Emerg Dis.

[16] Animal and Plant Health Agency (2015). Bovine tuberculosis: infection status in cattle in GB annual surveillance report for the period January to December 2014.

[17] Álvarez J, Bezos J, de la Cruz ML, Casal C, Romero B, Domínguez L (2014). Bovine tuberculosis: within-herd transmission models to support and direct the decision-making process. Res Vet Sci.

[18] McCorry T, Whelan AO, Welsh MD, McNair J, Walton E, Bryson DG (2005). Shedding of Mycobacterium bovis in the nasal mucus of cattle infected experimentally with tuberculosis by the intranasal and intratracheal routes. Vet Rec..

[19] Khatri BL, Coad M, Clifford DJ, Hewinson RG, Whelan AO, Vordermeier HM. A natural-transmission model of bovine tuberculosis provides novel disease insights. Vet Rec. 2012;171 10.1136/vr.101072 22935562

[20] Thom ML, Hope JC, McAulay M, Villarreal-Ramos B, Coffey TJ, Stephens S (2006). The effect of tuberculin testing on the development of cell-mediated immune responses during mycobacterium bovis infection. Vet Immunol Immunopathol.

[21] Drewe JA, O'Connor HM, Weber N, McDonald RA, Delahay RJ (2013). Patterns of direct and indirect contact between cattle and badgers naturally infected with tuberculosis. Epidemiol Infect.

[22] Conlan AJK, McKinley TJ, Karolemeas K, Pollock EB, Goodchild AV, Mitchell AP (2012). Estimating the hidden burden of bovine tuberculosis in Great Britain. PLoS Comput Biol.

[23] Birch CPD, Goddard A, Tearne O (2018). Bovine tuberculosis model for England and Wales (BoTMEW) including model description.

[24] Mitchell A, Bourn D, Mawdsley J, Wint W, Clifton-Hadley R, Gilbert M (2005). Characteristics of cattle movements in Britain - an analysis of records from the cattle tracing system. Anim Sci.

[25] Orton RJ, Bessell PR, Birch CPD, O'Hare A, Kao RR (2012). Risk of foot-and-mouth disease spread due to sole occupancy authorities and linked cattle holdings. PLoS One.

[26] Delahay RJ, Langton S, Smith GC, Clifton-Hadley RS, Cheeseman CL (2000). The spatio-temporal distribution of Mycobacterium bovis (bovine tuberculosis) infection in a high-density badger population. J Anim Ecol.

[27] Brunton LA, Nicholson R, Ashton A, Alexander N, Wint W, Enticott G (2015). A novel approach to mapping and calculating the rate of spread of endemic bovine tuberculosis in England and Wales. Spat Spatiotemporal Epidemiol.

[28] O'Hare A, Orton RJ, Bessell PR, Kao RR. Estimating epidemiological parameters for bovine tuberculosis in British cattle using a Bayesian partial-likelihood approach. Proc R Soc B. 2014;281 10.1098/rspb.2014.0248 PMC3996616 24718762

[29] Barlow ND, Kean JM, Hickling G, Livingstone PG, Robson AB (1997). A simulation model for the spread of bovine tuberculosis within New Zealand cattle herds. Prev Vet Med.

[30] Fischer EAJ, van Roermund HJW, Hemerik L, van Asseldonk M, de Jong MCM (2005). Evaluation of surveillance strategies for bovine tuberculosis (Mycobacterium bovis) using an individual based epidemiological model. Prev Vet Med..

[31] Beck K (2002). Test-driven development by example. Addison Wesley.

[32] Vernon MC (2011). Demographics of cattle movements in the United Kingdom. BMC Vet Res.

[33] Nuñez-Garcia J, Downs SH, Parry JE, Abernethy DA, Broughan JM, Cameron AR, et al. Meta-analyses of the sensitivity and specificity of ante-mortem and post-mortem diagnostic tests for bovine tuberculosis in the UK and Ireland. Prev Vet Med. 2017; 10.1016/j.prevetmed.2017.02.017 28347519

[34] Goodchild AV, Downs SH, Upton P, Wood JLN (2015). de la Rua-Domenech R. Specificity of the comparative skin test for bovine tuberculosis in Great Britain. Vet Rec.

[35] Trading options for farms under TB restrictions. *Better Returns programme.* Warwickshire: EBLEX, AHDB; 2014. https://beefandlamb.ahdb.org.uk/wp-content/uploads/2014/07/brpTB-restrictions080714a.pdf. Accessed 26/07/2018.

[36] Brooks-Pollock E, Conlan AJK, Mitchell AP, Blackwell R, McKinley TJ, Wood JL (2013). Age-dependent patterns of bovine tuberculosis in cattle. Vet Res.

