# Supplementary material for: A new bovine tuberculosis model for England and Wales (BoTMEW) to simulate epidemiology, surveillance and control
Source: BMC Vet Res. 2018 Sep 4;14:273. doi: 10.1186/s12917-018-1595-9 (PMC6122770; doi:10.1186/s12917-018-1595-9)
Supplement: Supplementary file 1 — Detailed functional description of a bovine tuberculosis model for England and Wales (BoTMEW), 41 pages. Part 1 Model specification. Part 2 Model setup and use. (DOCX 282 kb) [file 12917_2018_1595_MOESM1_ESM.docx]

**Detailed Functional Description of a Bovine Tuberculosis Model for England and Wales (BoTMEW)**

© Crown copyright

Authors: Colin P.D. Birch, Ashley Goddard and Oliver Tearne, Animal and Plant Health Agency (APHA)

Table of contents

**Part 1: The Model specification 3**

1. Overview 3
2. The holding 4
   1. Herd 4
   2. Environment 6
3. TB Transmission 6
   1. Within herd 6
   2. Environment to herd 7
   3. Changes to the environment 7
4. TB Transfer = cattle movements 8
   1. Matching movements to infected cattle 8
   2. Resolving potential movements of infected cattle 8
5. Surveillance 8
   1. Herd tests 9
   2. Individual tests, resulting from movements 10
   3. Counting tests 10
   4. False positives 11
6. Breakdowns 11
   1. Disclosing test and detection of lesions 12
   2. Resolution 12
   3. Execution 14

**Part 2: Model setup and use 15**

1. Cattle data 15
   1. Static characteristics of holdings 15
   2. Cattle movements 16
   3. Initial infection state of herds at the start of simulations 17
2. Parameter values 19
   1. Regions and time 19
   2. Unused parameters 23
   3. Set and fixed parameter values 23
   4. Parameter fitting 24
   5. Estimation of an upper 95% confidence limit for a *χ*^2^ based measure of model deviation from observed numbers of breakdowns. 24
   6. Estimates of the main parameters affecting infection in unrestricted herds 25
   7. Estimated parameter values affecting breakdown resolution 28
3. Environmental infection state 29
   1. Original setup of environmental infection state 29
   2. Adjustment to initial environmental infection state 29
   3. Initial set up of environmental infection states 33
   4. Fitting of the overall level of infection in environmental reservoirs 33
   5. Dynamics of infection in environmental reservoirs after 2010 33
4. Sensitivity analysis 34
   1. Transmission from environmental reservoirs 34
   2. Transmission from cattle 34
   3. Power law for density dependence 34
   4. Persistent infection 34
   5. Sensitivity and specificity of surveillance tests 36
   6. Confirmation and slaughterhouse surveillance 37
5. Outputs 37
   1. Summary outputs 37
   2. Breakdown outputs 39
   3. Final distribution of infected cattle 40
   4. Final infection state of environmental reservoirs 40
6. Anonymized model 41
   1. Anonymization 41
   2. Validation 41
7. References 41

**Part 1: The Model specification**

1. **OVERVIEW**

This description presents BoTMEW in two parts. This first part is a specification of its simulation of bovine tuberculosis (bTB) with its surveillance and control. The second part explains how it is set up and used.

BoTMEW is a network simulation model of *Mycobacterium bovis* transmission and control within England and Wales, at the resolution of farm holdings and individual infected cattle. It has time steps of a day for cattle movements and a month for evolution of infection. It focuses on the spread of *M. bovis* to individual holdings by import of infected cattle, or by transmission from a local environmental reservoir, which includes wildlife, as well as the detection of infected holdings by surveillance (Figure 1). The model also includes limited neighbourhood interactions representing transmission between environmental reservoirs. Transmission among cattle within herds is simulated, as well as the restrictions on herds detected as infected (‘breakdowns’), while they are being cleared of infection. However, these within herd processes are simplified compared with specifically within herd models (Conlan et al., 2012). Although there are several models of Foot and Mouth disease with scale and resolution comparable to BoTMEW (Dube et al., 2007), there has only been a single previous attempt to simulate bTB in a similar way (Brooks-Pollock et al., 2014), although other models have simulated bTB among large numbers of cattle holdings in imaginary spaces (Smith et al., 2016).

Figure 1: The processes that occur during simulation

1. **THE HOLDING**

The current version of the simulation is set in a spatially explicit network of cattle holdings derived from Cattle Tracing System (CTS) records for January 2008 to December 2010 (Mitchell et al., 2005), including every agricultural holding in England and Wales with a recorded cattle movement inward or outward, i.e. 73,371 holdings. Each holding consists of a herd of cattle and an associated environmental reservoir for *M. bovis* infection. CTS does not distinguish herds within a holding, so cattle at each holding are treated as a single herd.

- 1. Herd
     1. Static number of cattle and demography

The number of cattle at each holding is set as static throughout a simulation, calculated as being the average of the number of animals on the first day of each month during 2008-2010, excluding months when the holding is recorded as having no cattle. This simplification reduces the potential impact of input data errors during model runs, allows a simple, rigorous definition of the set of parameter values for each holding at any time, simplifies interpretation of model outputs and substantially reduces the volume of outputs required. Herds are linked to each other by cattle movements. The cattle in each herd are allocated to five age bins:

- 0-42 d: cattle too young to be tested for bTB.
- 43 d – 15 months: natural break associated with beef cattle slaughtered after their first year.
- > 15 – 30 months: natural break associated with beef cattle slaughtered after their second year.
- > 30 – 60 months: a break at 60 months divided the number of older cattle roughly evenly.
- > 60 months

These age ranges are referred to in the model description as ‘age bins’. Age is mainly used to constrain the movements of infected cattle; infection and detection of infection are assumed to be independent of the age of cattle.

- - 1. Dynamic number of infected cattle

*M. bovis* infection in the model is represented by the presence of individual infected cattle at holdings. Each cow infected with *M. bovis* is represented by the model as a distinct object. Each object carries with it the age of the cow and the day on which the cow was infected with disease; this allows at any time the calculation of the length of time that the cow has been infected. The age of an infected cow determines which age bin it is held in at a holding and which movements it may make.

When a cow is first infected, its age is determined in two steps. First its age bin is selected at random from the age bins at the holding, weighted by the number of cattle in each bin, and second by random generation from a uniform distribution within the age range of the selected bin. The maximum age bin does not have an upper age limit, so newly infected cattle in that bin will start at the minimum age of that bin, i.e. 60 months. This process assumes that age does not influence susceptibility to infection.

Infected cattle age each day, so they will occasionally need to be moved up to the next age bin at their holding. This updating process is the first operation performed each day, to ensure that cattle are in the correct age bins before any other daily processes occur.

Sometimes the simulation will require an infected cow to move, either by aging or by livestock movement, into a bin that has zero capacity, or that is already full of infected cattle. When this happens, the model randomly selects a cow (either from those already present or one moving into the bin) to be placed into an overflow ‘bucket’ associated with the age bin. Cattle in overflow buckets are given priority for future movements from their associated age bin and the adjacent age bins. The order of priority is shown in Figure 2. As well as avoiding potential technical conflicts, this process improves simulation of some movement processes. For example, a holding may be managed by regularly bringing in cattle at a particular age and moving them off at a specific older age. Because the model selects within age bins at random, by chance some animals may stay at the holding in the simulation longer than they would at the actual holding. The overflow process provides an automatic although delayed correction to the discrepancy between the simulation and actual animal management.


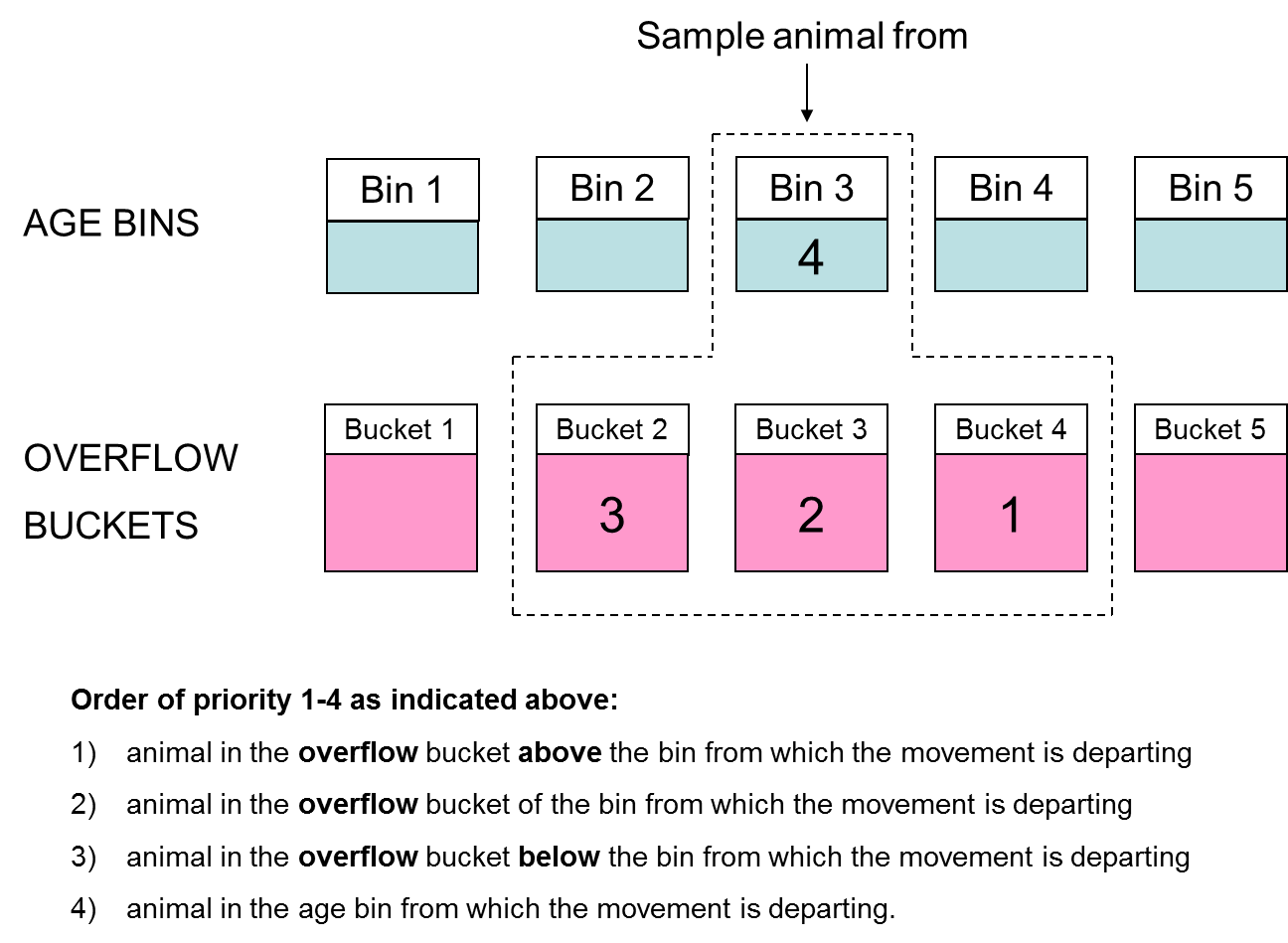


Figure 2: The order of priority of sampling animals from age bins and overflow buckets

- 1. Environmental reservoir

Apart from its cattle complement, the other information about a holding relates to its potential reservoir of environmental infection. Environmental infection in this model refers to prolonged persistence in the environment for at least a month and usually much longer, such as in wildlife. Transmission from cattle to cattle is mainly treated as if direct, even if it may have been via the environment with a short lag.

- - 1. Infection state of environmental reservoir

The infection state of the environmental reservoir can have five potential values:

- ‘X’: No potential for environmental infection. Cattle at the holding cannot be infected from environmental sources and the environmental infection state cannot change.
- ‘0’: There is no infection in the environmental reservoir. Cattle at the holding cannot be infected until the environmental infection state increases.
- ‘1’: The environmental reservoir has low level, reversible infection. Cattle at the holding are unlikely to be infected from the environment. Infection may either spread or become extinct in the environment.
- ‘2’: The environmental reservoir has established infection. Cattle at the holding may be infected from the environment, which is likely to remain infectious until controls are applied, e.g. badger controls.
- ‘3’: The environmental reservoir has temporarily heightened infection. This state may return to ‘2’ without intervention. Cattle at the holding have the highest risk of being infected from the environment.

Currently the values X and 3 have not been used and the value 2 is the only value at which the reservoir is infectious. The baseline scenario was too short to allow a significant impact from disease dynamics in environmental reservoirs. Therefore, during initial model fitting the environmental infection model was simplified by only using the two states 0 and 2.

1. ***M. bovis* TRANSMISSION**

The model applies a simple SI model in which cattle are either susceptible or infected with *M. bovis*. Once infected, cattle remain infectious until they are removed from the model, usually by detection and slaughter. Two processes generate new infected cattle within the model: transmission from infected cattle within herds, or transmission to cattle in a herd from their associated environmental reservoir.

- 1. Within herd transmission.

The within-herd transmission of *M. bovis* is assumed to be a monthly stochastic event where the number of new infections in a herd is sampled from a Poisson distribution with a mean of *λ* which is dependent on the number of infected and susceptible cattle in the herd and the transmission rate of *M. bovis*;

$$\lambda=\left( \frac{\beta I}{N^{q}} \right)S$$

where *I* = the number of infected cattle in the herd, *S* = the number of susceptible cattle on the holding, *N* = total herd size, *β* = the cattle to cattle transmission parameter and *q* = a power law determining the density dependence of transmission (*q* = 0 would match full density dependence, while *q* = 1 would match frequency dependence). The current version of the model assumes no lag before cattle become infectious, as assumed by the SOR model of Conlan, McKinley et al. (Conlan et al., 2012).

- 1. Transmission to cattle from their environment

The transmission of disease from the environment to cattle is also approximated as a monthly stochastic event in which new infections are estimated from a Poisson distribution with a mean of *λ*:

$$\lambda=\chi_{i}S$$

Where *S* = the number of susceptible animals on the holding and *h_i_* is the infection rate from the environment in state *i*. Currently *h_i_* = 0 unless *i* = 2.

- 1. Changes to infection in the environment

Implicit transmission of *M. bovis* to and among wildlife can also be simulated as changes between environmental states 0, 1, 2 and 3 using the transition matrix below, which is applied monthly (Table 1).

Table 1: Transition matrix for monthly changes to the infection state of environmental reservoirs.

|  | **From** | | | | |
| --- | --- | --- | --- | --- | --- |
| **To** |  | 0 | 1 | 2 | 3 |
|  | 0 | 1- *q*_1_ | *q_2_* := *p*_1_+ *k*_2_+ *k*_3_ | 0 | 0 |
|  | 1 | *q_1_*:*=k_1_n*(*I*) + $\sum_{i=1}^{3} a_{i}N(W_{i})$ | 1- (*q_2_* + *q_3_*) | *q_4_* := *k*_4_+ *k*_5_ | 0 |
|  | 2 | 0 | *q_3_* := *p*_2_ | 1-(*q_4_ + q_5_*) | *q_6_* := *p*_4_ |
|  | 3 | 0 | 0 | *q_5_* := *p*_3_ | 1- *q*_6_ |

Where *p_1_*, *p_2_*, *p_3_* and *p_4_* are probabilities for potentially spontaneous transitions, while *k_1_*, *k_2_*, *k_3_*, *k_4_*, *k_5_* are coefficients for determining the impact of sources of infection or control measures on the probability of a transition and *n*(*I*) is the number of infected cattle at the holding.

The sum term in the 0 to 1 transition formula represents potential spatial spread of wildlife TB infection to any holding from its six nearest neighbours, where *N*(*W_i_*) = the number of neighbours with wildlife index *i,* while *a_1_*, *a_2_* and *a_3_* are coefficients which determine the rate of the spatial spread of infection. This is the only spatial process in the model dependent on proximity. The six nearest neighbours are used as a crude method to identify holdings likely to be adjacent to each other without using data on the extent of holdings: polygons in a regular array will mostly be adjacent to 6 neighbours, as in a hexagonal grid (Birch, 2006). Actual farms are rarely in regular arrays, but the average number of adjacent neighbours will tend to increase rather than decrease as farm areas become more complex. If a different number of nearest neighbours was used the main impact would be to modify the values of the parameters *a_i_*.

1. ***M. bovis* TRANSFER = CATTLE MOVEMENTS**

Because the cattle composition of every holding is static, the only role of cattle movements is to transfer *M. bovis* infection between holdings. Cattle movements were collected from the Cattle Tracing Scheme (CTS) database for 1^st^ January 2008 to 31^st^ December 2010. Each movement record details the source and destination holdings, and the number of cattle to be moved from each of the five age bins. In addition to farm-to-farm movements this data set also contains movements to slaughter and movements to holdings located in Scotland. Holdings within Scotland are not explicitly included in the model and thus any movements to Scotland are recorded as animals that have left the system. In principle Scotland could be included in the model by extending the holdings and movements input and ignoring ‘Scotland’ as a destination, or using it for movements out of Great Britain.

- 1. Matching movements to infected cattle

Movements are drawn from the movement data every day, but each movement is ignored unless:

- - 1. The origin holding includes infected cattle. AND
    2. The origin holding is not under restriction. AND
    3. The movement includes cattle from an age bin that includes infected cattle at the source holding.
  1. Resolving potential movements of infected cattle

If the movement satisfies all three conditions, the movement is checked for whether it includes infected cattle. As shown in Figure 2, any infected cattle in the overflow buckets of the age bin moving, or adjacent age bins, are moved first. Then any remaining cattle in the movement may be taken from the matching age bin. The selection is resolved by sampling cattle one at a time without replacement, so, if the number of cattle moving equals or exceeds the total number of cattle in an age bin, all infected cattle in the age bin move. If infected cattle are moved, pre-movement tests are applied for holding to holding and holding to Scotland movements, if required at the origin holding. Undetected cattle are then moved to their destinations, or removed and recorded as transferred to Scotland. For holding to slaughter movements, infected cattle are removed and slaughterhouse surveillance is applied.

1. **SURVEILLANCE**

The model distinguishes five surveillance streams for detecting *M. bovis* infection in unrestricted cattle. Three of these are herd tests resolved monthly, while the remaining two streams are individual tests resolved after movements of infected cattle. Any reactors detected by these surveillance streams immediately trigger breakdowns at the holdings.

In all surveillance streams, the duration of infection (or time since infection) is a factor that influences the probability of detection. This approach allows the probability that an infected animal will be detected to change over time in a different way for different surveillance streams, as well as unlimited regional variations, while only requiring a single variable to be maintained for the infected animal. Changes in test sensitivity over time are represented as “ramp” functions: sensitivity increases linearly with duration of infection until a maximum sensitivity is reached.

- 1. Herd tests
     1. Regular herd tests

Regular herd tests are herd bTB tests applied at regular intervals to all cattle herds, at a frequency determined mostly by the herd’s location (before 2013, the “parish testing interval”). They consist of

- “Whole herd tests”, which test most animals in a herd in regions with annual or biennial tests.
- “Routine herd tests”, which test about half the animals in a herd, excluding young animals and those that will not be kept to reproductive age.

The model simulates this process by applying a surveillance test at a fixed interval determined by the current control regime. Within the model the interval between regular herd tests is referred to as the herd test interval (HTI). Holdings in breakdown or waiting for a post-breakdown test are not available for regular herd tests.

When a herd is tested, the animals to be tested are randomly selected from the infected animals within each age bin *i* and associated overflow bucket with equal, age dependent probability = *w_i_*. When an infected animal is selected for testing it is detected with probability

$$p=\text{MIN}\left( a_{r},b_{r}t \right)$$

where *t* = the time in days for which the animal has been infected, *a_r_* = the maximum sensitivity of the test and *b_r_* = the rate at which the test increases sensitivity per day.

- - 1. Post breakdown test

Post breakdown tests are applied to herds 6 months (the “6 month” test) and 18 months (the “12 month” test) after the end of a breakdown. They postpone the cycle of regular herd tests after the end of a breakdown. Herds in breakdown are not available for post breakdown tests. When a post breakdown test occurs, each infected animal on the holding is detected with probability

$$p=\text{MIN}\left( a_{b},b_{b}t \right)$$

where *t* = the time in days for which the animal has been infected, *a_b_* = the maximum sensitivity of the test and *b_b_* = the rate at which the test increases sensitivity per day.

- - 1. Generic herd test

The surveillance for bTB within England and Wales includes many surveillance streams that are not explicitly simulated by the model. The generic herd test implemented in the model is a monthly abstraction representing the combined effect of all the surveillance not explicitly simulated by the model. Many of the surveillance streams are targeted, so the probability that herds receiving generic herd tests are infected may be greater than predicted assuming random sampling. This targeted surveillance may increase the ratio of infected reactors relative to the number of tests and the number of false positive reactors.

Every month herds not in breakdown are randomly selected for generic testing with probability *g_i_* if infected and probability *g_u_* if not infected. The *g_i_* and *g_u_* parameters represent the targeting of herd tests, so *g_i_* is likely to be greater than *g_u_*. Within herds selected for generic testing, each infected animal is detected with probability

$$p=\text{MIN}\left( a_{g},b_{g}t \right)$$

where *t* = the time in days for which the animal has been infected, *a_g_* = the maximum sensitivity of the test and *b_g_* = the rate at which the test increases sensitivity per day.

- 1. Individual tests, resulting from movements
     1. Pre-movement test

Within regions in which pre-movement testing is applied, when a movement of an infected animal between holdings or to Scotland takes place, all infected animals selected for movement are subject to a pre-movement test. All movements from the holding that day are cancelled if any infected animals are detected. Infected animals within a movement are detected with probability

$$p=\text{MIN}\left( a_{mv},b_{mv}t \right)$$

where *t* = the time in days for which the animal has been infected, *a_mv_* = the maximum sensitivity of the test and *b_mv_* = the rate at which the test increases sensitivity per day. These parameters take account of pre-movement test exemptions, which are not explicitly applied in the model.

- - 1. Slaughterhouse surveillance

When infected animals are moved to slaughter they are subject to slaughterhouse surveillance, which detects them with probability

$$p=\text{MIN}\left( a_{s},b_{s}t \right)$$

where *t* = the time in days for which the animal has been infected, *a_s_* = the maximum sensitivity of the test and *b_s_* = the rate at which the test increases sensitivity per day. Infected animals that are not detected by slaughterhouse surveillance are recorded as having left the model undetected through slaughter.

- 1. Counting tests

The model gathers information on the numbers of animal tests carried out to allow quantification of the costs of control. The numbers of tests also determine the numbers of false positive reactors. The count of animal tests increases by $\sum_{i=1}^{5} w_{i}n_{i}$ for each regular herd test, where *w_i_* = the proportion of animals tested and *n_i_* = the number of cattle in each age bin *i* of the herd*.* For each post breakdown herd test, the count of animal tests increases by the number of animals in the herd, which may be an over-count, because animals younger than 42 d are not tested and herds may have less animals than average after a breakdown. Also, for each generic herd test, the count of animal tests increases by the number of animals in the herd. The model’s count of pre-movement tests = the number of movements from herds that are within regions with pre-movement testing. The model does not apply pre-movement test exemptions, so the count of pre-movement tests is an overestimate that should be corrected outside the model.

- 1. False positive test results

False positive test results are generated monthly from the counts of animal tests in each herd. Each surveillance stream *x* has its specificity *Sp_x_*, so the number of false positives is:

$$\sum_{\text{all} x} \text{B}\left[ n_{x}, 1-{Sp}_{x} \right]$$

where B[*n,r*] = a binomial variable equivalent to *n* trials with probability *r* and *n_x_* = the number of animal tests during the month in surveillance stream *x*. It is assumed that slaughterhouse surveillance does not generate false positives. Specificities should be adjusted for any expected over-counting of tests, especially pre-movement tests.

1. **BREAKDOWNS**

When a reactor or slaughterhouse case is detected by any of the surveillance streams included in the model, the holding at which the detection was made immediately enters the breakdown process. The breakdown process prevents the holding from moving cattle to other holdings until the holding meets criteria for considering the breakdown resolved, which are intended to leave the holding with a high probability of being clear of infected cattle. In reality the breakdown is a process that develops over at least a number of months. However, the holding is isolated as a source of infection during the breakdown and it is assumed that most infected cattle present will be detected by the end of the breakdown. Therefore, to simplify model structure and parameterization, focus model development on epidemiology and surveillance in unrestricted herds, and make the model robust to changes in procedures during breakdown resolution, the model resolves a new breakdown immediately at its start (Figure 3). In this section any unreferenced quantitative statements are based on Colin Birch’s calculations from APHA’s VetNet TB data during development of this model.

Figure 3: Factors influencing a breakdown outcome (left) and outputs resolved at the start of a breakdown (right).

- 1. Disclosing test and detection of lesions

The first step in resolving the breakdown is to decide whether the disclosing test detected reactors confirmed by visible lesions or culture of *Mycobacterium bovis*. Breakdowns including confirmed reactors are assumed to be managed as if “Officially tuberculosis free status withdrawn” (OTFW), while other breakdowns are managed as “Officially tuberculosis free status suspended (OTFS). All infected animals present at the start of the breakdown are provisionally considered to be detected and are checked to see if lesions are detected.

Cases detected by slaughterhouse surveillance are by definition treated as confirmed by lesions, so the breakdown is OTFW. Otherwise lesions are detected in infected animals, including the first case, with probability *p*.

$$p=MIN(a_{L},b_{L}t_{I})$$

where *a_L_* is the probability that lesions are found in reactors that have been infected long enough to have maximum probability of having visible lesions, *b_L_* is a coefficient determining the probability that lesions will be found in reactors before infection has persisted long enough to reach maximum probability of having visible lesions and *t_I_* is the length of time for which the animal has been infected. If at least one animal, including the first detected animal, is found to have lesions, the breakdown becomes an OTFW breakdown. If the breakdown includes at least one reactor (i.e. not a slaughterhouse case) without detected lesions, apart from the disclosing case, the breakdown may end with an undetected infected animal still present (see below). Note that the model assumes that the presence of detectable lesions, infectiousness, reactivity to skin tests and detection by other surveillance tests are independent, except through their association with time since infection.

- 1. Breakdown resolution
     1. Determination of breakdown duration

The breakdown length is determined by *SIT*, an integer number of short interval tests. To ensure a minimum length for the breakdown, a constant is added *M*, which during 2008-2010 was 1 short interval test for OTFS breakdowns *M_U_* and 2 short interval tests for OTFW breakdowns *M_C_*. Hence:

*SIT = M + T*

*Duration* (months) = 2 *SIT +* 1

Where *T* is the number of additional short interval tests beyond *M*. The extra month added to duration reflects the tendency for intervals before short interval tests to be longer than the minimum 60 days. For example, the median duration of an OTFS breakdown during 2002 - 2010 was 84 days.

Preliminary examination of the duration of breakdowns found that the duration had a significant linear relationship with the logarithm of the number of reactors in the disclosing test, which explained only a small part of the variation in breakdown length. The overall distribution of breakdown length was like a geometric distribution with an extended tail. Breakdown length was also strongly related to herd size, as observed by Conlan et al. (2012). Therefore the proposed model for *T* is that it is a variable generated as the sum of a Poisson distributed variable and a variable with a Geometric distribution:

*T =*Poisson(*k_I_* log(*n_I_*)) *+* Geometric(1/(*k_w_nh_i_+k_n_n+k_B_*+1))

Where *n*, *n_I_* and *h_i_* are the number of cattle in the herd, the number of cattle infected at the start of the breakdown, and the local wildlife infection rate, as above. While *k_I_, k_w_* and *k_n_* are three regional control parameters. Finally *k_B_* has different values in OTFW breakdowns *k_C_* and OTFS breakdowns *k_U_*. A value of *T =* 0 would imply that there were no reactors in a breakdown after the disclosing test.

The definition of the Geometric distribution varies, so, for clarity, we have used the form:

P[*X=y* | *X*=Geometric(*p*)] = *p*(1*-p*)*^y^* for all *X* ≥ 0

To use the alternative form ( *p*(1*-p*)*^y-^*^1^ *X* ≥ 1), the values of *M* should be reduced by 1.

- - 1. Number of short interval surveillance tests in a breakdown

It is assumed that all animals on a holding are tested at every short interval test. Hence the number of animal tests during the breakdown is:

*Numtests = n SIT*

where *n* is the number of cattle in the herd.

- - 1. Number of reactors in a breakdown

The model assumes that all infected cattle present at the start of a breakdown will be detected, except a single animal may remain undetected (see below). If the duration of the breakdown is beyond minimum *T* > 0, additional animals may also become infected and detected during the breakdown. The number of additional reactors is assumed to be a Poisson variable:

If *T =* 0, *n_Bv_ =* *n_Bnv_* = *n_Bs_* = 0

Else *n_B_ =* Poisson(*m_I_ n_I_ + m_w_h_i_ nT*)

Where *T* is the number of additional short interval tests from 6.2.1.

*n_B_ =* the number of additional reactors occurring and detected during the breakdown.

*n*, *n_I_* and *h_i_* are as defined above.

*m_I_*, and *m_w_* are two more regional control parameters.

Additional reactors may have visible lesions, but the duration of infection is unknown. Therefore the probability that each extra reactor has visible lesions is set as constant for each control region = *p_L_*. If an OTFS breakdown has any additional reactors with lesions or slaughterhouse cases, its status changes to OTFW, but no adjustment is made to calculations already completed for the breakdown.

- - 1. False positive reactors in a breakdown

The large number of short interval tests during breakdowns potentially generate false positive reactors.

*n_F_ =* Poisson(*p_F_ nT*)

Where *p_F_* = (1- the test specificity). The value of *p_Fc_* for OTFW breakdowns may substantially differ from *p_Fu_* for OTFS breakdowns, because sensitivity of tests during breakdowns may be increased at the expense of reduced specificity. The equation uses *nT* rather than *Numtests* because the final test(s) must be clear.

- - 1. Persistence of infection in a herd after a breakdown ends

If a breakdown includes at least one reactor without detected lesions, apart from the first detected animal (“disclosing case”), there is a chance that infection persists in one animal at the holding after the end of the breakdown. Slaughterhouse cases cannot be counted for this condition. As an approximation, the number of persistent animals is limited to one because, if it is assumed that all test results are independent, the holding is much more likely to end the breakdown with 0 or 1 infected cattle present than with more than 1 (Conlan et al., 2012), although in reality more than one infectious animals sometimes persist at the end of breakdowns, which will affect onward infection dynamics. Assuming at least one reactor qualifies, the potentially persistent infected animal is an additional reactor generated as described in 6.2.3 without detected lesions. If there were no additional reactors without lesions, the potentially persistent infected animal is the most recently infected animal present at the start of the breakdown, apart from the disclosing case. Persistence depends on herd size, occurring with probability:

$$p_{P}=\mathrm{MIN}\left(c_{P}+m_{P}n,q_{P}\right)$$

where *c_P_,* *m_P_* and *q_P_* are regional control parameters. Additional reactors that persist beyond the end of a breakdown have their infection duration at the end of the breakdown set at:

*t_I_* = *d_P_* days

Where *d_P_* is a regional control parameter.

If the persistent infected animal was present at the start of the breakdown, its age and duration of infection should be increased by the duration of the breakdown. If the persistent infected animal is an additional reactor, it should have its age at infection determined by the same process as is used for any other newly infected animal. Its age at the end of the breakdown should be its age at infection plus its duration of infection.

- 1. Breakdown execution

During a breakdown, all cattle movements from the holding are cancelled. All infected animals attempting to move to a breakdown holding are given pre-movement tests if required. If they are not detected they return to or remain at their source holding. No transmission of *M. bovis* occurs to or among cattle at a breakdown holding, because any transmission is implicit in the calculation of the number of additional reactors. Nor is there any transmission from cattle at a breakdown holding to other cattle or to the local environmental reservoir. The model does not simulate the number of infected cattle present during a breakdown, so any transmission it generated would be arbitrary, and it was important to avoid transmission of *M. bovis* to the environment from breakdowns started by false positive reactors. No surveillance tests are applied to breakdown holdings apart from short interval tests as described above.

The breakdown ends at the start of the month *Duration* months after the start of the breakdown. This approach to rounding to the beginning of a month will tend to add a few more days to breakdowns starting from herd tests than to breakdowns starting from pre-movement tests or slaughterhouse surveillance.

After the breakdown ends, post breakdown tests should be applied 6 months (6 month test) and 18 months after the breakdown (12 month test). After a negative 12 month test, the holding returns to the regional regular herd test interval.

As implied above, if an infected animal persists at the end of a breakdown, it has no opportunity to move or transmit infection until the day the breakdown ends. Thus the infected animal can first transmit infection within the holding on the day the breakdown ends. Movements off the holding, which may or may not include the infected animal, will also resume on this day.

**Part 2: Model setup and use**

Although the main aim is to explain how the model works and was developed, this section aims to provide minimal although sufficient explanation to allow a reader to run, modify and examine outputs from an example of the model provided with input files for the baseline scenario. The current release is v0.8.8. In the root folder the files “run_windows.bat”, “ReleaseNotes.txt”, “log4j.properties” and “configProperties.txt” should be visible, with the folders “initialisation”, “lib” and “util”; the folder “output” is generated when the model starts. The model can be started by selecting “run_windows.bat” and double clicking, or pressing return. Model runs can be modified by editing “configProperties.txt” and the contents of “initialisation”.

1. **CATTLE DATA**

There is an imbalance in the data available on bTB in Great Britain. In contrast to the dearth of data on bTB in the environment and wildlife, there are abundant data available on observed bTB in cattle, as well as on cattle movements that may transfer *M. bovis* between herds. These data are nearly complete throughout Great Britain since 2002. Therefore the model used direct data for setting the denominator population of cattle and herds, the initial state of *M. bovis* in cattle, and for representing transfer of *M. bovis* by cattle movements. Set up is described as if for the baseline model fitted to data from 2008 – 2010, but it is hoped that readers can see how equivalent procedures can be used for any alternative set up.

- 1. Static characteristics of holdings

The list of holdings in the model and their static characteristics are taken from the Cattle Tracing System (CTS) records for January 2008 to December 2010 (Mitchell et al., 2005), which included 73,371 holdings with at least one recorded cattle movement inward or outward. These are listed in the input file “SE3122_Holdings_ Numeric.csv” with the fields:

- Hold = A unique integer ID number for each holding arbitrarily defined during model development.
- Easting and Northing = the 6 digit Ordnance Survey coordinates in units of metres. These are not used in the current version of the model, so they are set to 000000 in the anonymized version of the model.
- Type = Beef, dairy, mixed or other. The model does not currently use this field.
- Five fields for the average number of cattle in each age bin, averaged across the first day of each month during 2008 – 2010 when the holding contained at least one cow.

Another input file “SE3122_Holdings_CPH_lookup.csv” contains only two fields:

- Hold = The integer ID number as above.
- CPH = The 9 digit county / parish / holding identifier for each holding. In the anonymized version of the model the parish and holding references are replaced with values derived from the ID number.

The input file “NearestNeighbours.csv” lists for each holding ID number the ID numbers of its six nearest neighbouring holdings.

- 1. Cattle movements

The time frame of the model is in effect driven by the table of cattle movements entered into it. The model starts running from the first date in the movement data at least to the end of the movement data. If the model is required to run beyond the last recorded movement date, selection of movements restarts from the beginning of the movement data. This cycling of movements is controlled by settings within the file “configProperties.txt”:

- simulationStartDate = the date of the earliest movements in the cattle movement data.
- movements.yearsInLoop = the number of years of movement data provided
- movements.timesToLoop = the number of times the movements should be repeated, so the simulation duration is the product of these two settings.

The recycling of movements implies two assumptions:

- The population of holdings and their cattle composition continue the same for the extended period.
- The movement records collected are representative of movements that would take place during the extended time.

The model is constructed as in Figure 1 so that the process of executing cattle movements is separate from other tasks. This allows movements to be replayed on a daily basis while some other processes can be performed less frequently.

The file “SE3122_MvtsNoReplacement_Numeric.csv.txt” provides a list of all cattle movements during the simulation in order of date. Movements were extracted from the Cattle Tracing System (CTS) records for 1st January 2008 to 31^st^ December 2010 (Mitchell, Bourn et al. 2005). Each movement includes all the cattle moving between a specific pair of holdings in one day, and is described by a single record (row) including:

- Date = The day on which the movement took place.
- Off = The holding ID from which the cattle moved
- On = The holding to which cattle moved, “73372” indicates Scotland, while “73373” indicates movement to slaughter.
- Age_... = Five fields for the number of cattle moving in each of the five age ranges defined in Part 1, paragraph 2.1.1.
  1. Initial infection state of herds at the start of simulations
     1. Herds waiting for post breakdown tests

The file “RecentBD.csv” lists holdings that had their most recent breakdowns ending less than 18 months before 1^st^ January 2008. This input identifies which holdings will have post breakdown tests early in the simulation and when, and includes the fields:

- CPH = The CPH ID of the holding where the breakdown took place.
- Month = The month when the holding is next due a post breakdown test.
- Test-type = The test that will be applied (6 months after the breakdown or 18 months).
  - 1. Herds in breakdowns

The file “CurrentBD.csv” lists all holdings that were in breakdown at the time the simulation starts. This input identifies which holdings are restricted at the start of the simulation and when their restrictions will end:

- CPH = The CPH ID of the holding where the breakdown is taking place.
- Exit_Month = The month when the breakdown will end.
  - 1. Initial herds containing undetected infected cattle <3b>

Herds that started actual breakdowns a few days after 1^st^ January 2008 were very likely to already contain infected cattle on that date. This was less true for herds that started breakdowns months later, but the chronological list of herds starting breakdowns on or after 1^st^ January 2008 provided a first approximation to a list of herds that included undetected infected cattle that day. However, it is uncertain which breakdowns actually involved infected animals. For the 2008-2010 scenario, all OTFW breakdowns were assumed to include actual infected animals, as well as all breakdowns at holdings that had at least one other breakdown during 2002-2010. Conversely, OTFS breakdowns that were the only breakdown at their holding during 2002-2010 were assumed to result from false positive reactors. The list also only retains the first breakdown at each holding after 1^st^ January 2008, and excludes breakdowns at holdings that were restricted on 1^st^ January 2008. The input file “FutureBD.csv” includes all breakdowns from 1/01/2008 to 31/12/2009 with the fields:

- CPH = The CPH ID of the holding of the breakdown.
- Day = The day when the breakdown took place.
- Num_infected = The number of reactors detected at the disclosing test.
- Type = The surveillance test that triggered the breakdown: a herd test, pre-movement test or slaughterhouse surveillance.
- Confirmed = Whether the actual breakdown included confirmed reactors: Y (Yes) or N.
- Initialisation = Whether the simulation will potentially treat this holding as starting the simulation with undetected infected cattle: T (True) or F.
- Holding_type = Dairy, beef or “other”.
- Test_interval = The herd test interval of the herd at the time of the breakdown.

The number of herds starting with undetected infected cattle is controlled by the setting “breakdownsToInitialise” in “configProperties.txt” (current baseline value = 4400). Herds are selected in chronological order of breakdown date, starting with 1/01/2008. This setting was adjusted during simulation runs until it was at a value at which the total number of breakdowns in 2008 was inside the range 4850 – 5050, which was within about 100 of the observed number (4949).

- - 1. Initial numbers of undetected infected cattle

In each herd identified as starting a simulation with undetected infected cattle, the initial number of undetected infected cattle was

$1+\frac{\left(t_{Z}-t_{i}\right)\left(m_{i}-0.5\right)}{t_{Z}}$ rounding down.

where *t_Z_* is the observed time from the start of the simulation to the latest actual breakdown date of all the holdings that initially included undetected infected cattle, *t_i_* is the day on which breakdown *i* occurred and *m_i_* is the actual number of reactors at the breakdown’s disclosing test.

The formula given above approximates by assuming all infections start with a single bovine (hence +1), that all infections took *t_Z_* days to detect, and that infection had a constant rate within each breakdown (hence $(t_{Z}-t_{i})m_{i}/t_{Z}$). The simulation is assumed to start midway between when the most recent bovine became infected and when the next one will become infected (hence *m_i_* – 0.5).

If it is determined that only one infected animal should be present on the holding at initialisation, it is assumed to have been infected for length of time $t_{Z}-t_{i}$. Each additional infected animal that is initialised at the same holding has the length of time it has been infected for reduced in increments of $t_{Z}/(m_{i}-0.5)$. The aim was to introduce some variety into the initial state of simulations, based on plausible dependencies, e.g. the number of undetected infected cattle in herds would be correlated with the number detected at the eventual breakdown, and would be inversely correlated with the delay until the breakdown started.

1. **PARAMETER VALUES**

BoTMEW potentially has an almost unlimited number of distinct parameter values. Every parameter file includes 63 parameters (Table 2), which can all be given values differing between regions and months. Regions are geographic units defined as sets of holdings, so it is possible although very impractical to provide different parameter values for every individual holding. The model was designed to be very flexible in its parameter setting because it was likely to be used to investigate unforeseen control options in previously undefined geographical regions. Holdings must also be given initial settings determining the probability that they start with *M. bovis* infection in their environmental reservoirs, because, while the initial state of cattle and their infection were based on abundant data, there is in effect no localized data on *M. bovis* infection in wildlife or contamination of the environment. In practice many parameter values were set equal, and relatively few were varied across regions or time. Here the parameter values are presented with the evidence, reasoning and processes used to set them. Unless stated otherwise, parameter values are quoted from a baseline model scenario starting on 1/01/2008, fitted to breakdowns reported in 2010, then projected forward to the end of 2016, with some parameters modified locally or nationally after 2010 to represent changes in bTB surveillance and control.

- 1. Regions and time
     1. Every holding belongs to a single region defined in the file “CPH_Region_ Lookup.csv”, which provides a text name for the region including each CPH ID. Therefore regions cannot overlap: if an overlap region is required that combines the properties of region “A” and region “B” it must be identified as a new region, perhaps with a name like “AB”.
     2. The file “ControlRegionCalendar.csv” must have a row for every distinct region name in “CPH_Region_ Lookup.csv”, and a column for every month of the simulation run. For every month in every region it provides the name of the parameter file that lists the 63 parameter values that should be applied. Different regions can share the same parameter files at the same or different times. Each parameter file is a plain text file listing 63 parameters, with the variable names used to identify them in the model’s computer code and their values for the regions and months referring to the file.
     3. Since 2013 herd test intervals and other controls have been mainly aligned within Wales and three bTB management areas in England. Contiguous counties in North and East England with low bTB incidence are designated as the ‘Low Risk Area’ (LRA), in contrast to the ‘High Risk Area’ (HRA) in the South-west and a buffer zone in between (‘Edge’) (2014). Hence regions in the baseline set up of BoTMEW are subdivisions of the four management areas: Wales, HRA, LRA and Edge.

Table 2: Basic information on parameters listed in parameter files in the order listed. Section numbers in parentheses in the Description field indicate sections of Part 2 of the Model Description that provide further information about the parameters. Range shows the range of values used in a baseline simulation running from 2008 – 2016, including geographical variation across England and Wales as well as temporal variation. Range is left blank if only a single value was used; commas separate a small number of different values; a dash separate lowest and highest values.

| Name in  Parameter file | Description | Symbol in text | Value, PTI1 2010 | Range |
| --- | --- | --- | --- | --- |
| fromCattleParameters.beta | Cattle to cattle transmission rate within herd (2.6.1) | *β* | 0.008 mth^-1^ | 0.008, 0.011 mth^-1^ |
| fromCattleParameters.q | Power law for density dependence of transmission (2.6.1) | *q* | 0.5 | - |
| wildlifeParameters.k1 | Transmission rate from cattle to wildlife at holding (3.3). | *k*_1_ | 0 | 0, 0.10 mth^-1^ |
| wildlifeParameters.k2 | Extinction of environmental *M. bovis* from infection state 1 due to badger culling. Not used (2.1.1). | *k*_2_ | 0 | - |
| wildlifeParameters.k3 | Extinction of environmental *M. bovis* from infection state 1 due to badger vaccination. Not used (2.1.1). | *k*_3_ | 0 | - |
| wildlifeParameters.k4 | Reduction of environmental *M. bovis* from infection state 2 to 1 due to badger culling or stochastic change (3.3). | *k*_4_ | 0 | 0, 0.036 mth^-1^ |
| wildlifeParameters.k5 | Reduction of environmental *M. bovis* from infection state 2 to 1 due to badger vaccination. Not used (2.1.1). | *k*_5_ | 0 | - |
| wildlifeParameters.p1 | Stochastic extinction of environmental *M. bovis* from infection state 1 (2.3.3). | *p*_1_ | 0.5 mth^-1^ | - |
| wildlifeParameters.p2 | Stochastic recovery of environmental *M. bovis* from infection state 1 to 2 (2.3.3). | *p*_2_ | 0.5 mth^-1^ | - |
| wildlifeParameters.p3 | Stochastic increase of environmental *M. bovis* from infection state 2 to 3. Not used (2.1.1). | *p*_3_ | 0 | - |
| wildlifeParameters.p4 | Stochastic decrease of environmental *M. bovis* from infection state 3 to 2. Not used (2.1.1). | *p*_4_ | 0 | - |
| fromBadgerParameters.X0 | Environment to cattle transmission rate at infection state 0. Zero by definition. (2.1.3) | *h*_0_ | 0 | - |
| fromBadgerParameters.X1 | Environment to cattle transmission rate at infection state 1. Not used (2.1.1). | *h*_1_ | 0 | - |
| fromBadgerParameters.X2 | Environment to cattle transmission rate at infection state 2. | *h*_2_ | 0.0007 cow^-1^ mth^-1^ | 0.00021, 0.0007 |
| fromBadgerParameters.X3 | Environment to cattle transmission rate at infection state 3. Not used (2.1.1). | *h*_3_ | 0 | - |
| lesionParameters.aL | Maximum proportion of reactors confirmed (2.6.3). | *a_L_* | 0.4 | - |
| lesionParameters.bL | Increase in proportion of reactors confirmed per day infected (2.6.3). | *b_L_* | 0.011 d^-1^ | - |
| preSlaughter.aS | Maximum sensitivity of slaughter surveillance (2.6.4). | *a_S_* | 0.875 | 0.557 – 0.875 |
| preSlaughter.bS | Increase in sensitivity of slaughter surveillance per day infected (2.6.4). | *b_S_* | 0.025 d^-1^ | 0.0037 – 0.025 d^-1^ |
| preMovement.testing | Flag for pre-movement surveillance (2.3.2) | *-* | TRUE | TRUE, FALSE |
| preMovement.aSk | Maximum sensitivity of a pre-movement test (2.6.5). | *a_mv_* | 0.32 | - |
| preMovement.bSk | Increase in sensitivity of a pre-movement test per day infected (2.6.5). | *b_mv_* | 0.009 d^-1^ | - |
| preMovement.sP | Specificity of a pre-movement test (2.6.5). | *Sp_mv_* | 0.9999 | - |
| regularTest.aR | Maximum sensitivity of a regular herd test (2.6.5). | *a_r_* | 0.48 | 0.45, 0.48 |
| regularTest.bR | Increase in sensitivity of a regular herd test per day infected (2.6.5). | *b_r_* | 0.014 d^-1^ | 0.013, 0.014 d^-1^ |
| regularTest.w1 | Proportion of cattle in age bin 1 tested in a regular herd test. (2.1.4) | *w*_1_ | 0 | - |
| regularTest.w2 | “ in age bin 2 “ (2.6.6). | *w*_2_ | 0.78 | 0.5, 0.64, 0.78 |
| regularTest.w3 | “ in age bin 3 “ (2.6.6). | *w*_3_ | 0.78 | 0.5, 0.64, 0.78 |
| regularTest.w4 | “ in age bin 4 “ (2.6.6). | *w*_4_ | 0.78 | 0.5, 0.64, 0.78 |
| regularTest.w5 | “ in age bin 5 “ (2.6.6). | *w*_5_ | 0.78 | 0.5, 0.64, 0.78 |
| regularTest.sp | Specificity of a regular herd test (2.6.5). | *Sp_r_* | 0.9998 | - |
| postBreakdown.aB | Maximum sensitivity of a post breakdown test (2.6.5). | *a_b_* | 0.53 | 0.45, 0.53 |
| postBreakdown.bB | Increase in sensitivity of a post breakdown test per day infected (2.6.5). | *b_b_* | 0.015 d^-1^ | - |
| postBreakdown.sP | Specificity of a post breakdown test (2.6.5). | *Sp_b_* | 0.9998 | - |
| persistence.cP | Minimum probability that a qualifying breakdown retains persistent infection at its end (2.6.7). | *c_p_* | 0.25 | 0.1, 0.125, 0.25 |
| persistence.mP | Additional probability that a qualifying breakdown retains persistent infection at its end per cow in herd (2.6.7). | *m_p_* | 0.00116 | 0.00022 – 0.00116 |
| persistence.qP | Maximum probability that a qualifying breakdown in a large herd retains persistent infection at its end (2.6.7). | *q_p_* | 0.6 | 0.3, 0.5, 0.6 |
| persistence.dP | Assumed duration of infection in a persistent infected cow at the end of a breakdown if infected during the breakdown (2.6.7). | *d_p_* | 50 d | - |
| genericTest.a | Maximum sensitivity of a generic herd test (2.6.5). | *a_g_* | 0.48 | 0.45, 0.48, 0.53 |
| genericTest.b | Increase in sensitivity of a generic herd test per day infected (2.6.5). | *b_g_* | 0.014 d^-1^ | 0.013 - 0.015 d^-1^ |
| genericTest.gi | Proportion of infected herds receiving a generic herd test (2.6.6). | *g_i_* | 0.02 mth^-1^ | 0.02 - 0.09 |
| genericTest.gu | Proportion of uninfected herds receiving a generic herd test (2.6.6). | *g_u_* | 0.014 mth^-1^ | 0.0012 – 0.04 |
| genericTest.sp | Specificity of a generic herd test (2.6.5). | *Sp_g_* | 0.9998 | - |
| breakdownDuration.kI | Coefficient relating breakdown duration to initial number of reactors (2.7.1). | *k_I_* | 0.5 log(*n_I_*)^-1^ | 0, 0.5 log(*n_I_*)^-1^ |
| breakdownDuration.kW | Coefficient relating breakdown duration to environmental *M. bovis* (2.7.1). | *k_W_* | 6.0 | - |
| breakdownDuration.kN | Coefficient relating breakdown duration to herd size (2.7.1). | *k_n_* | 0.001 cow^-1^ | 0.0005, 0.001 |
| breakdownDuration.kU | Constant additional breakdown duration of OTFS breakdowns (2.7.1). | *k_U_* | 0.57 | - |
| breakdownDuration.kC | Constant additional breakdown duration of OTFW breakdowns (2.7.1). | *k_C_* | 1.20 | - |
| breakdownDuration.mU | Minimum duration of OTFS breakdown (2.3.2) | *M_U_* | 1 | 1, 2 |
| breakdownDuration.mC | Minimum duration of OTFW breakdown (2.3.2) | *M_C_* | 2 | - |
| breakdownDuration.offset | Additional duration of breakdown beyond minimum test intervals (2.3.3) | *-* | 1 mth | 1 mth |
| breakdownReactors.mI | Coefficient relating number of additional reactors in a breakdown to its initial number of reactors (2.7.2). | *m_I_* | 0.389 | 0.346, 0.389 |
| breakdownReactors.mW | Coefficient relating number of additional reactors in a breakdown to environmental *M. bovis* (2.7.2). | *m_W_* | 7.632 | 6.784, 7.632 |
| breakdownReactors.mT | Coefficient relating number of additional reactors in a breakdown to breakdown duration. Not used (2.1.5). | *m_T_* | 0 | - |
| breakdownReactors.mSc | Coefficient relating number of slaughterhouse cases in an OTFW breakdown to breakdown duration. Not used (2.1.2). | *m_Sc_* | 0 | - |
| breakdownReactors.mSu | Coefficient relating number of slaughterhouse cases in an OTFS breakdown to breakdown duration. Not used (2.1.2). | *m_Su_* | 0 | - |
| breakdownReactors.pL | Proportion of reactors in a breakdown that are confirmed (2.7.3). | *p_L_* | 0.4 | 0.1, 0.3, 0.4 |
| breakdownFalsePositive.pFu | Proportion of tests in OTFS breakdowns that result in false positive reactors (2.7.3). | *p_Fu_* | 0.00047 | 0.00047 - 0.0016 |
| breakdownFalsePositive.pFc | Proportion of tests in OTFW breakdowns that result in false positive reactors (2.7.3). | *p_Fc_* | 0.0009 | 0.009 – 0.0009 |
| neighbours.a1 | Transmission to holding environment from each neighbour environment at infection state 1. Not used (2.1.1). | *a*_1_ | 0 | - |
| neighbours.a2 | Transmission to holding environment from each neighbour environment at infection state 1 (3.3). | *a*_2_ | 0 | 0, 0.01 |
| neighbours.a3 | Transmission to holding environment from each neighbour environment at infection state 1. Not used (2.1.1). | *a*_3_ | 0 | - |
| testInterval | Interval between regular herd tests (2.3.2) | *-* | 12 mth | 6-48 mth |

- 1. Unused parameters

Currently 15 of the parameters in the parameter files are always set equal to zero (Table 2).

- - 1. This is mostly because they are required by model features that have not been used yet or are not in use, such as a transition to represent super-shedding from badgers in the environment.
    2. Parameters relating to slaughterhouse cases during breakdowns are set to zero because the cases are relatively unimportant for bTB dynamics, and data on such cases was not readily accessible during model development.
    3. Environmental infection state 0 is intended to indicate that there is no environmental source of infection.
    4. Currently surveillance tests are not applied to cattle in age bin 1, which is age less than 42 d.
    5. The parameter relating additional reactors in a breakdown to its duration is set equal to zero to avoid generating infected cattle in the absence of a source of infection in breakdowns started by a false positive surveillance test.
  1. Set and fixed parameter values
     1. The file “configProperties.txt”, which is kept with the “run_windows.bat” file that starts model simulation runs, contains several parameters that control model runs:
- the number of undetected infected herds present at the start of simulations (section 1.3.3 above),
- settings to control the length of simulations and the movement data they use (section 1.2 above).
- “simulation.numRuns” sets the number of times the simulation is repeated.
- “simulation.minimumIndex” sets the integer index for the first simulation of the series, with the index increasing one for each successive rerun.
- “binUpdate.frequency” controls the model’s management of overflow of cattle age bins (Part 1, section 2.1.2).
- Finally three flags set the outputs produced by the model, whether to generate outputs for breakdowns during simulations, and for infected cattle remaining and infection state of the environment at the end of simulations.
  - 1. Four parameters in the parameter file did not require estimation because they simply define a current local control measure (Table 2). One is a flag to indicate whether pre-movement tests are required. Another states the local herd test interval. Another two parameters define the local minimum number of short interval tests in OTFW and OTFS breakdowns, which are set by regulations (Part 1, section 6.2.1).
    2. Three more parameters in the parameter files are used to control model behaviour (Table 2). Currently the model is set to avoid using environmental infection state 1, which represents very low infection levels: when the environmental reservoir has recently become infected, or when infection is close to extinction. The two parameters representing establishment of infection or extinction of infection are both set equal to 0.5, so an environmental reservoir that enters infection state 1, either through exposure to infection from cattle, or through reduction from infection state 2, will change again to infection state 0 or 2 at the start of the next month. Another parameter simply ensures that breakdown duration is one month longer than the minimum duration to complete its number of short interval tests (Part 1, section 6.2.1).
  1. Parameter fitting

The complexity of the model and the large and indefinite number of different parameter values put formal identification of optimal values using fully automated algorithms beyond the resources available during model development. Instead, sets of parameter values achieving good fits were identified by using external parameter estimates, applying model constraints and through iteration, comparing simulations with different parameter estimates informed by previous simulation outputs. Because there was indefinite scope for improvement and there were alternative criteria for evaluating model fit, the set of parameter values used for sensitivity analysis and model exploration is referred to as the ‘baseline’ set of parameter values (Table 2). The original baseline was fitted to breakdowns reported in 2010, after starting on 1/01/2008.

A $\chi_{24}^{2}$measure of deviation of the model from the actual number and distribution of breakdowns in 2010 was calculated by comparing for each of the four bTB management areas the numbers of breakdowns from the five surveillance streams and the number of OTFW breakdowns, i.e 6 x 4 = 24 comparisons. The number of breakdowns for comparison with the model was slightly below the official statistics, because breakdowns were only relevant if they occurred at holdings included in the model and the model only has one herd per holding. The $\chi_{24}^{2}$measure was the sum of 24 terms of the form:

$\chi^{2}=\frac{\left(N_{\text{model}}-N_{\text{observed}}\right)^{2}}{N_{\text{model}}}$ (3)

where *N*_model_ is the average number of breakdowns from model runs. This measure was expected to have an approximately *χ*^2^_24_ distribution, so it is referred to as the ‘*χ*^2^_24_ value’. An important criterion for estimating the main parameters of the model was to minimize the *χ*^2^_24_ value.

- 1. Estimation of an upper 95% confidence limit for a *χ*^2^ based measure of model deviation from observed numbers of breakdowns.

*χ*^2^_24_ measures of model deviation from observed numbers of breakdowns were calculated from 200 replicates of a simulation with a baseline set of parameter values. Using the cumulative density function of the Binomial distribution, there is greater than 95% confidence that 95% of all equivalent measures would have a value equal to or less than the highest value exceeded by no more than 5 out of 200 replicates, which was 151.8. The mean value of the *χ*^2^_24_ measure was 101.0, so, with 95% confidence, the difference between the mean value and the 95 percentile was no more than 50.8.

In any comparison between the model and observed values we compare a single observed instance with a known number of model replicates. If the number of model replicates was very large we might choose to reject the model fit if the average *χ*^2^_24_ measure of model deviation exceeded 151.8. The equivalent threshold for rejection when comparing 10 model replicates with observed values would be 101.0+50.8*√(1+1/10) = 154.3.

- 1. Estimates of the main parameters affecting infection in unrestricted herds

The primary interest of this model is in infection processes in unrestricted herds, while the presence of infection in herds is undetected. However, because infection processes depend on the unknown levels of infection in cattle and environmental reservoirs, parameters were mainly estimated by iterative fitting. Parameters are discussed in the order they are listed in Table 2, omitting parameters that were set zero up to 2010, or which mainly affect outputs from breakdowns.

- - 1. Cattle to cattle transmission

Cattle to cattle transmission was determined by a transmission rate *β* and a power law parameter *q* as in Part 1, section 3.1 above. The power law was set to the intermediate value *q* = 0.5 following the conclusion of Conlan, McKinley *et al.* (2012) that 0 < *q* < 1 but they could not precisely estimate its value. The transmission rate *β* was fitted to match the observed numbers of reactors once the model was achieving a reasonable match to the observed numbers of breakdowns. However, fitting was obscured by the unknown distinction between cattle infected while herds were unrestricted and cattle infected during breakdowns. Model fitting started from the central estimate of *β* ≈ 0.013 mth^-1^ from Conlan, McKinley *et al.* (2012). The original value after fitting to 2010, given *q* = 0.5, was *β* = 0.008 mth^-1^, but this seemed to underestimate numbers of reactors when projected forward to 2016, so the estimate for 2011 and later was increased to *β* = 0.011 mth^-1^. In a herd of 200 cattle, this would be equivalent to a frequency dependent transmission rate of 1.70 yr^-1^, which, in a herd of that size, would be relatively high compared with the estimates of density dependent rates reported by Álvarez, Bezos et al. (2014), but low compared with the frequency dependent rates.

The drift in the value of *β* need not be surprising. We have no evidence that transmission rate for *M. bovis* remains constant with time (Álvarez et al., 2014), and no strong reason for expecting transmission rate to be a robust constant. It may be sensitive to changes in management practices, the weather, or any other aspect of the system. Alternatively, the increased number of reactors requiring the adjustment to transmission rate might have been due to another unidentified change in the system, such as a change in skin test sensitivity (Downs et al., 2013). The impact of any change on the estimate of *β* would be amplified by a negative feedback: the increase in the number of reactors from increasing *β* is reduced by the increased sensitivity of surveillance at herd level as the number of infected cattle is increased.

- - 1. Environment to cattle transmission

In the absence of badger controls, the model assumes that all cattle exposed to infectious environmental reservoirs (infection state 2) have the same individual probability *h* of becoming infected each month. The estimate for this transmission rate strongly interacted with the estimate of the number of holdings with infectious environmental reservoirs, as explained in section 3 below.

- - 1. Confirmation of reactors

The maximum probability that an infected reactor was confirmed *a_L_* was relatively easily estimated, because the only major output affected by it was the number of OTFW breakdowns. Although the model assumed all reactors were equally likely to be confirmed, confirmed reactors were actually unevenly distributed among breakdowns, so this estimate, to match the proportion of breakdowns that were OTFW, was lower than the overall proportion of infected reactors that were confirmed.

Model outputs were insensitive to the rate at which lesions developed. Moreover, Brooks-Pollock, Conlan et al. (2013) reported that the peak rate of confirmation of reactors was in cattle less than 2 years’ old, coinciding with the start of the age range at which age-specific bTB incidence was maximum. These observations could be consistent with visible tuberculous lesions developing early in infection if they develop at all. Therefore the rate of increase in confirmation rate *b_L_* was set to be equivalent to maximum confirmation rate being reached 35 d after infection, matching the rate set for the development of surveillance test sensitivity below.

- - 1. Slaughter surveillance

Surveillance at slaughter detects infection in cattle that have already left all other parts of the management and surveillance system, so its sensitivity *a_S_* is relatively easily estimated because it has little impact apart from increasing the number of breakdowns detected at slaughter. From 2008 to 2010 the sensitivity of slaughter surveillance appeared to increase, coinciding with a programme of additional training in bTB for FSA meat inspectors (Broughan et al., 2014). To match the observed number of breakdowns detected by slaughter surveillance, its sensitivity in 2010 was estimated to be substantially higher than the proportion of reactors confirmed. Several factors may have contributed to this apparent anomaly.

- The rate of confirmation of reactors in Table 2 is an underestimate at the resolution of individual cattle. (See 2.5.3 above.)
- The peak proportion of reactors with visible lesions is at ages of about 2 years (Brooks-Pollock et al., 2013), which is within the age range at which most slaughter takes place.
- Breakdowns at holdings under some styles of management not simulated by the model, including approved finishing units, are very likely to start with slaughterhouse cases.

Similarly, Nuñez-Garcia et al. (2017) reported high estimates of sensitivity of meat inspection relative to ante-mortem surveillance tests and rates of confirmation of test reactors.

The rate of increase of the sensitivity of slaughter surveillance *b_S_* was set to be equivalent to maximum sensitivity being reached 35 d after infection, matching the time to maximum confirmation rate (2.5.3).

- - 1. Surveillance tests

Most surveillance tests applied to unrestricted herds in Great Britain are Single Intradermal Comparative Cervical Tuberculin (SICCT) skin tests (Nuñez-Garcia et al., 2017). Their specificities *Sp_r_*, *Sp_b_*, *Sp_g_* and *Sp_mv_* were based on the estimate of Goodchild et al. (2015), with the specificity of pre-movement tests increased to compensate for the model applying them too frequently.

It has been difficult to estimate the maximum sensitivity *a_r_*, *a_b_*, *a_g_* and *a_mv_* of the SICCT skin test as applied in the field (Nuñez-Garcia et al., 2017), but recent estimates have tended to be lower than previous estimates (Álvarez et al., 2014). The estimate here at around 0.5 was similar to the value recently reported by Nuñez-Garcia et al. (2017), but it should be considered very uncertain. The sensitivity of pre-movement tests was reduced to compensate for the model applying them too often. To take account of the potential for subjective judgement, the sensitivity of tests in areas with low bTB incidence was slightly reduced, while the sensitivity was slightly increased for post breakdown tests in areas with high bTB incidence.

Model behaviour was insensitive to the values set for the rate at which sensitivity increased with the duration of infection *b_r_*, *b_b_*, *b_g_* and *b_mv_*, so these parameters were set to a rapid rate of increase based on experimental evidence that cattle inoculated intranasally could not be detected by the skin test for up to 2 weeks after inoculation, but were consistently detected at 3 weeks and beyond (Thom et al., 2006). Values were set to be equivalent to surveillance tests reaching full sensitivity at 35 d.

- - 1. Numbers of surveillance tests

The parameters *w*_1_ – *w*_5_ set the proportion of cattle in each age bin tested in a regular herd test. These proportions differed between herd test intervals and management areas because regular herd tests in the model represented a mix of different actual herd tests at regular intervals. “Whole herd tests”, which include most cattle in a herd, are usual under annually testing, while “routine herd tests”, which exclude many cattle not used for breeding, are usual under less frequent testing. The proportions were also adjusted to improve the model’s match to the observed numbers of regular herd tests, noting that herd management may result in herds tending to be below their average size when they are tested.

As a simple way to represent targeted testing, the probability of generic herd tests differed between infected herds *g_i_* and uninfected herds *g_u_*. The value of *g_u_* was fitted to match the number of surveillance tests, while *g_i_* was fitted to match the numbers of breakdowns starting with tests represented by generic herd tests. These parameters were relatively likely to differ between parameter files because various specific control measures were represented using the generic herd tests.

- - 1. Persistence of infection at the end of breakdowns

A possible explanation for infected cattle being detected in a herd soon after it has cleared a breakdown is a failure to detect one or more infected cattle persisting in the herd when the breakdown ended. For infection to persist in the model, a breakdown must satisfy the preconditions explained in Part 1, section 6.2.5. The parameters *c_p_* the minimum probability that infection will persist in a small herd, *m_p_* the additional probability per head in the herd and *q_p_* the maximum probability that infection will persist were based on the outputs of Conlan et al. (2012). These parameters were relatively likely to differ between parameter files because breakdowns were a focus of many additional control measures, which often aimed to reduce persistence of infection at the end of breakdowns. For example, use of interferon gamma blood tests could reduce persistence by detecting a higher proportion of infected cattle.

Cattle infected during a breakdown and persisting beyond its end required a duration of infection at the end of the breakdown *d_p_*. No data could be available to inform this parameter and its precise value was not expected to have a substantial impact on model behaviour. If it was less than 60 d, the infected cow had implicitly persisted after passing only one short interval test, but some infected cattle may be missed by two or more successive tests. A value of 50 d therefore seemed a plausible estimate of an average value.

- 1. Estimated parameter values affecting breakdown resolution

The procedures for managing bTB breakdowns are complicated and they have multiple outputs, so the model required over 10 parameters to specify their representation. However, in the model most of the outputs from breakdowns have little impact on the continuation of the bTB epidemic, so accurate estimation of these parameters was given lower priority than parameters affecting bTB in unrestricted herds.

- - 1. Breakdown duration

On the one hand, the duration of a breakdown was a cost, due to its restriction of farm management and the costs of disease control. On the other hand, herd restrictions removed sources of infection from the cattle movement network. Coefficients relating breakdown duration to the initial number of reactors present *k_I_*, herd size *k_n_* and whether the breakdown was confirmed *k_C_* or unconfirmed *k_U_* were estimated from analysis of breakdowns recorded from 2002-2011. The coefficient for the effect of infection from the holding’s environmental reservoir on breakdown duration *k_W_* was set equal to 6.0 because exploration with a simple model suggested that the expected number of extra short interval tests was at least six times the monthly probability that at least one cow would be infected from the environmental reservoir. During model development it was found that including breakdown duration was necessary to fit the model, but the model was not very sensitive to the precise duration of breakdowns. For example, increasing the value of *k_W_* from 6.0 to 20.0 in the baseline model decreased the total number of confirmed breakdowns in 2016 from 3096 to 2805 (averages from 10 simulations).

- - 1. Number of reactors in a breakdown
    2. The number of additional reactors infected during a breakdown is related to the initial number of reactors present by the coefficient *m_I_* and the potential for infection from the environmental reservoir by the coefficient *m_W._* These were calculated to match the baseline model to total numbers of reactors in 2010. The values in Wales were set at 8/9 times the values in England to reduce a difference in the model deviation between Wales and England. The number of reactors in a breakdown is only a model output, with no effect on any other process or output in or from the model. So far the number of reactors has not been a priority output from model applications. Confirmation and false positive reactors

The proportion of additional infected reactors that are confirmed *p_L_* is set at up to the value (0.4) for the proportion of reactors from surveillance tests that are confirmed *a_L_*. It is set at lower values of 0.3 in the Edge and 0.1 in the LRA to represent the lower proportions of reactors that are confirmed in those regions. False positive reactors can potentially arise at each short interval test in a breakdown. The proportion of short interval tests in OTFS breakdowns that generate false positive reactors *p_Fu_* may be lower than the proportion in OTFW breakdowns *p_Fc_*, because test interpretation is often more severe in OTFW breakdowns. Numbers of false positive reactors may also be higher when interferon gamma tests are used.

- 1. Sensitivity analysis

The *χ*^2^_24_$\chi_{24}^{2}$ value was used to indicate potential ranges for individual parameter values by observing the impact on the average *χ*^2^_24_$\chi_{24}^{2}$ value of varying parameters from their baseline values (Tables 1 and 2). Based on the conservative argument of Supplementary text Annex 4, an average *χ*^2^_24_$\chi_{24}^{2}$ measure from 10 model replicates greater than 154.3 was treated as a significant deterioration of model fit at P < 0.05. The workbook in the supplementary material ‘Comparison.xlsx’ contains the numbers of observed breakdowns in 2010 to 2012 that were compared with model outputs, and illustrates how the *χ*^2^_24_ value was generated directly from model outputs from 10 simulations using the baseline set of parameter values.

1. **ENVIRONMENTAL INFECTION STATE**

The initial fitting was within a three year scenario from the beginning of 2008 to the end of 2010, which was too short for model outputs to be substantially affected by changes in the distribution of infection in wildlife. The trend during 2003-2010 was for OTFW bTB incidents to increase c. 8% per year, but the underlying increase per herd tested has been much slower (Animal Health and Veterinary Laboratories Agency, 2010). Therefore only wildlife states ‘0’ and ‘2’ were used, with no simulation of transitions between wildlife states. When simulations were projected forward to 2016 some dynamics were introduced to infection in environmental reservoirs, balancing potential introduction from infected cattle and transfer among neighbouring environments with a chance of spontaneous loss of infection from environmental reservoirs.

- 1. Original setup of environmental infection state

After discussion with epidemiologists at APHA, the definitions in Table 3 were used to classify eight wildlife regions with probabilities that holdings had infectious wildlife present at the start of simulations. These definitions were influenced by the target of matching the numbers of recurrent breakdowns observed, given an observation from pilot runs of early versions of the model that at least 70% of breakdowns in the HRA were caused by transmission from wildlife.

- 1. Adjustment to initial environmental infection state

The initial classification of Table 3 and the resulting distribution of infectious environmental reservoirs at holdings were used as first estimates in an iterative process. It was noted that this classification required development, because it lacked a geographic component and relied too heavily on details of TB history. The model was fitted to match observed numbers of breakdowns in 2010 by adjusting other parameters. Matching the observed numbers of breakdowns in the LRA was prioritized, so that the model could be assumed to be correctly estimating the number of breakdowns not directly due to infection from wildlife. Then the distribution of OTFW breakdowns in 2010 among counties was compared with observed numbers. This identified the deviation between the number of OTFW

Table 3: Definitions of the original eight different wildlife regions for simulations starting on 1/01/2008.

| **Class** | **Description** |
| --- | --- |
| 1 | Wildlife state = 0.   - In a Low incidence county - Or less than two TB incidents at holding during last 5 years. - And no past incident ending after 1/07/2006. - And no breakdown after 1/01/2008. - And local bTB incidence < 1 reactor / 1000 animals / year, or in an Edge county. |
| 2 | Probability 0.4 that Wildlife state = 2, else Wildlife state = 0.   - Less than two TB incidents at holding during last 5 years - TB incident ending between 1/07/2006 and 31/10/2008, or breakdown after 1/01/2008 but not potentially infected on 1/01/2008. - In an Edge county |
| 3 | Probability 0.2 that Wildlife state = 2, else Wildlife state = 0.   - No TB incidents at holding during last 5 years, nor any breakdown after 1/01/2008. - Local TB incidence ≥ 1 reactor / 1000 animals / year. - In a High incidence county or Wales |
| 4 | Probability 0.5 that Wildlife state = 2, else Wildlife state = 0.   - Less than two TB incidents at holding during last 5 years - Current breakdown on 1/01/2008 ending after 31/10/2008, or potentially infected on 1/01/2008. - In an Edge county |
| 5 | Probability 0.6 that Wildlife state = 2, else Wildlife state = 0.   - Less than two TB incidents at holding during last 5 years - TB incident ending between 1/07/2006 and 31/10/2008, or breakdown after 1/01/2008 but not potentially infected on 1/01/2008. - In a High incidence county or Wales |
| 6 | Probability 0.7 that Wildlife state = 2, else Wildlife state = 0.   - Less than two TB incidents at holding during last 5 years - Current breakdown on 1/01/2008 ending after 31/10/2008, or potentially infected on 1/01/2008. - In a High incidence county or Wales. |
| 7 | Probability 0.6 that Wildlife state = 2, else Wildlife state = 0.   - Less than two TB incidents at holding during last 5 years - And no past incident ending after 1/07/2006. - And no breakdown after 1/01/2008. - Local TB incidence ≥ 1 reactor / 1000 animals / year. - In a High incidence county or Wales. |
| 8 | Wildlife state = 2   - Two or more TB incidents at holding during last 5 years - In a High incidence or Edge county. |

breakdowns from the model and the number observed for each county. The deviation was treated as significant if

$\left(N_{\text{model}}-N_{\text{observed}}\right)^{2}>2N_{\text{model}}$ (3.2.1)

which approximated a significant difference at the 95% level of confidence, assuming the number of breakdowns was Poisson distributed.

Two of the model outputs were the number of cattle in unrestricted herds infected from wildlife in each county and the number infected from other cattle. Where the number of OTFW breakdowns significantly differed between the model and observation, these outputs were adjusted by multiplying by the ratio of the number of observed OTFW breakdowns in each county to the average number of OTFW breakdowns from the model. Hence the number of cattle in unrestricted herds infected from the environment in 2010 was estimated for each county. Significant evidence that a county, or a group of neighbouring counties contained infectious environmental reservoirs required

$N_{\text{wildlife}}^{2}>2\left(N_{\text{cattle}}+N_{\text{wildlife}}\right)$ (3.2.2)

where *N_wildlife_* and *N_cattle_* were the adjusted model estimates of the number of infections from wildlife and cattle, applying a similar test and assumptions to Equation 3.2.1. This criterion indicated that the northern regions of Wales, Gwynedd and Clwyd, Nottinghamshire in England and the whole of the current LRA could be treated as including no infectious environmental reservoirs during 2008-2010. Moreover, in other counties, it was assumed that holdings with recent local cattle TB incidence rate for SICCT test reactors below 0.001 yr^-1^ had zero probability of having infectious environmental reservoirs, unless they had a TB breakdown ending between July 2006 and the end of 2010.

Hence the number of infections from environmental reservoirs to unrestricted cattle and the number of cattle at holdings that might have infectious environmental reservoirs were identified for each county. This allowed an estimate to be made for each county of the incidence of TB infection from environmental reservoirs to cattle per head of cattle exposed to infectious environmental reservoirs. To reduce the number of classes of different incidences, these estimates were grouped into four clusters by value with averages: 0.00073 yr-1, 0.00121 yr-1, 0.00170 yr-1, and 0.00232 yr-1. Counties within each cluster were not necessarily close to each other geographically.

In addition, within county variation in the probability that holdings had infectious environmental reservoirs was represented. Holdings were identified as having a higher probability of an infectious environmental reservoir if they were among holdings at the start of simulations expecting a post breakdown test, or initially seeded with infected cattle: i.e. if they were in breakdown during 1/07/2006 – 31/12/2007, if they had an OTFW breakdown during 1/01/2008 – 31/12/2010, or if they had an OTFS breakdown during 1/01/2008 – 31/12/2010 and another breakdown during 2002-2010. Hence holdings were classified into nine different values for the probability that they had infectious environmental reservoirs: the value zero and two non-zero values for each of four groups of counties (Table 4).

Allowing breakdown history after the start of the simulation to influence the distribution of environmental reservoirs was necessary because detection of recurrent breakdowns was explicitly simulated (i.e. breakdowns detected by post breakdown tests), while undetected infections present at the start of simulations were seeded using data about later breakdowns. Since infection from environmental reservoirs was potentially a major cause of recurrent breakdowns, their distribution had to be substantially correlated with the distribution of undetected infected cattle at the start of each simulation. Seeding undetected infected cattle and infectious environmental reservoirs independently would have prevented the simulation from generating enough recurrent breakdowns.

Table 4: Counties with numbers of holdings in the model with zero and non-zero probabilities of having infected wildlife reservoirs

|  |  | Number of holdings with zero or non-zero probabilities of having infected environmental reservoirs | | |
| --- | --- | --- | --- | --- |
| County | ID | Zero | Non-zero probabilities | Non-zero Total |
| Avon | 34 | 1 | P = 0.1 : 730 ; P = 0.45 : 214 | 944 |
| Berkshire | 02 | 320 | P = 0.828 : 2 | 2 |
| Buckinghamshire | 03 | 687 | P = 0.184 : 14 | 14 |
| Cheshire | 06 | 1583 | P = 0.1 : 333 ; P = 0.45 : 65 | 398 |
| Cornwall | 07 | 132 | P = 0.1 : 2229 ; P = 0.45 : 1057 | 3286 |
| Derbyshire | 09 | 1127 | P = 0.1 : 823 ; P = 0.45 : 181 | 1004 |
| Devonshire | 10 | 97 | P = 0.14 : 3695 ; P = 0.63 : 1807 | 5502 |
| Dorset | 11 | 457 | P = 0.062 : 884 ; P = 0.279 : 210 | 1094 |
| Dyfed | 55 | 521 | P = 0.062 : 3608 ; P = 0.45 : 1129 | 4737 |
| East Sussex | 41 | 739 | P = 0.14 : 39 ; P = 0.63 : 19 | 58 |
| Glamorgan | 57-59 | 659 | P = 0.14 : 319 ; P = 0.63 : 38 | 357 |
| Gloucestershire | 14 | 1 | P = 0.184 : 799 ; P = 0.828 : 561 | 1360 |
| Gwent | 60 | 10 | P = 0.1 : 668 ; P = 0.45 : 274 | 942 |
| Hampshire | 15 | 1203 | P = 0.828 : 5 | 5 |
| Hereford and Worcs. | 17 | 18 | P = 0.184 : 1430 ; P = 0.828 : 913 | 2343 |
| Leicester | 22 | 1250 | P = 0.184 : 37 | 37 |
| Northamptonshire | 29 | 750 | P = 0.184 : 12 | 12 |
| Oxfordshire | 33 | 585 | P = 0.14 : 96 ; P = 0.63 : 35 | 131 |
| Powys | 52 | 550 | P = 0.14 : 1589 ; P = 0.63 : 590 | 2179 |
| Shropshire | 35 | 910 | P = 0.184 : 1054 ; P = 0.828 : 450 | 1504 |
| Somerset | 36 | 904 | P = 0.062 : 1675 ; P = 0.279 : 419 | 2094 |
| Staffordshire | 37 | 405 | P = 0.14 : 1622 ; P = 0.63 : 495 | 2117 |
| Warwickshire | 43 | 487 | P = 0.14 : 250 ; P = 0.63 : 69 | 319 |
| West Midlands | 46 | 141 | P = 0.14 : 17 ; P = 0.63 : 1 | 18 |
| Wiltshire | 45 | 319 | P = 0.14 : 733 ; P = 0.63 : 333 | 1066 |
| Other counties |  | 27992 |  |  |
| Total |  | 41848 |  | 31523 |

Fitting indicated that, to generate enough breakdowns at post-breakdown tests, a large contrast in probability was required within counties between holdings with a higher probability of having infected environmental reservoirs and holdings with lower probability. Therefore, the higher probability in each county was set to 4.5 times the lower probability, which set the highest baseline probability for a holding to have an infectious wildlife reservoir at 0.828, which allowed some scope in sensitivity analyses for increasing probabilities above their baseline values (Table 4). Note that all counties included at least one holding with zero probability of having an infected environmental reservoir, although such holdings were a small minority in several counties.

- 1. Initial set up of environmental infection states

The input file “WildlifeCodeMatrix.csv” provides a table in which each of the nine levels of potential infection in environmental reservoirs are set probabilities for starting simulations in each of the possible infection states, so that the sum of probabilities for each level of infection equals one. In the baseline set up probabilities are only above zero for state 0 = not infected and state 2 = infectious. The input file “WildlifeCodes.csv” defines which of the nine levels of infection is applied to every CPH in the model.

- 1. Fitting of the overall level of infection in environmental reservoirs

For model fitting, the relative distribution of initial infection level was maintained, while varying the total number of holdings with infectious environmental reservoirs. This was achieved by summarising the overall burden of infection from wildlife as *N_W_,* the expected number of holdings with infectious environmental reservoirs in England and Wales. The value of *N_W_* could be changed by increasing or decreasing all probabilities of reservoirs being infectious by the same multiple, effectively modifying the values of the probabilities in Table 4, but maintaining their allocation to counties.

- 1. Dynamics of infection in environmental reservoirs after 2010

The baseline model was established for 2008-2010 with static environmental reservoirs to simplify model fitting, given that environmental change could have little impact within 3 years, and changes might not be observed within such short time periods (Delahay et al., 2000). Later, some potential for change in the distribution of infected environmental reservoirs was introduced into simulations projected beyond 2010. The aim was to allow holdings with infected environmental reservoirs to establish a more natural clustering, as well as allow interactions between impacts on disease in cattle and disease in the environment. Within the HRA, Edge area and Wales, the probability of infection transmitting from cattle at a holding to its environmental reservoir *k*_1_ was increased from 0 to 0.1 infected cow^-1^ mth^-1^ (Table 2). (Because the probability that infection established in the environment from state 1 to state 2 = 0.5, this was equivalent to a probability of the environmental reservoir becoming infectious = 0.05 infected cow^-1^ mth^-1^.) Although this was intended to be a generous setting, it seemed to only generate small numbers of new infectious environmental reservoirs, possibly because most infected cattle were at holdings that already had infectious environmental reservoirs. Therefore the probability that infection would transmit from infectious environmental reservoirs to reservoirs at their 6 nearest neighbour holdings *a*_2_ was also increased from 0 to 0.01 per infectious neighbour holding mth^‑1^.

These two sources for fresh infection of environmental reservoirs were balanced by increasing the probability *k*_4_ that environmental reservoirs would spontaneously reduce from infection state 2 to state 1, which had a probability 0.5 of becoming extinct, from 0 to 0.036 mth^-1^. Fitting suggested that the net effect of these three changes on the total burden of infection in environmental reservoirs was negligible.

1. **SENSITIVITY ANALYSIS**

To help understand the behaviour of the model, as well as to guide iterative changes while fitting parameters, the impact of 9 of the most important model parameters on 9 model outputs, as well as on the measure of deviation was investigated. The sensitivity analysis is summarised by a multiplot (Figure 4), which demonstrates the biological factors constraining acceptable parameter values. It also illustrates the correlations among the effects of changes in parameter values on outputs.

- 1. Transmission from environmental reservoirs

Transmission from the environment was a major driver of TB breakdowns, being critical for matching the excess of breakdowns in the HRA relative to the LRA. It could be regarded as having two components: the number of holdings exposed to infectious wildlife reservoirs *N_W_* and the transmission rate from infectious wildlife *h*. Figure 4 shows that the effects of varying these two parameters were collinear, so that a large value of *N_W_* could compensate for a small value of *h* and vice versa.

- 1. Transmission from cattle

While transmission from wildlife was the main determinant of the number of breakdowns, transmission from cattle determined the number of cattle infected (Figure 4). The number of cattle infected in the LRA was especially sensitive to cattle to cattle transmission, because cattle to cattle transmission strongly affected the number of infected movements into the LRA as well as its direct effect within the LRA. Therefore the value of the transmission rate parameter *β* was mainly determined by the number of cattle infected and the contrast between the HRA and LRA in the number of cattle infected, rather than the numbers of breakdowns.

- 1. Power law for density dependence

Despite investigating the full range from 0.0 to 1.0 of the power law for the degree of density dependence of cattle to cattle transmission *q*, the parameter was found to have relatively little impact (Figure 4). The only substantial impact of changing this parameter was to increase the number of infected cattle in the LRA if it was reduced below 0.5.

- 1. Persistent infection

In contrast, breakdowns ending with infected cattle undetected (called here ‘persistent infection’) could generate substantial numbers of breakdowns, especially breakdowns detected by post breakdown tests (Figure 4). Thus persistent infection

Figure 4: An array of miniplots to demonstrate the sensitivity of the model to changes around the baseline set of parameter values. Each column is a parameter being varied. Each row is an output measure. Within the miniplots, axes cross at the baseline values of parameters and outputs, while the vertical axis is the output value as a multiple of its baseline average, from x0.7 to x1.5. Horizontal axes are individually defined by column as (minimum-baseline-maximum). Columns are from left to right: Envt. Holdings = Number of holdings with infectious environmental reservoirs; Envt. rate = Environment to cattle transmission rate; Cattle = Cattle to cattle transmission rate in a herd of 200 cattle; Power = Power law for degree of frequency dependence of cattle to cattle transmission; Persist = Proportion of possible breakdowns that have persistent infection at their end; Sensitivity = sensitivity of the standard skin test; Specific = specificity of the standard skin test; Confirm = Probability of confirming an infected reactor; Slaughter = sensitivity of slaughterhouse surveillance. Rows are from top to bottom: Deviance = model deviance; Cattle = Number of cattle infected while in unrestricted herds in the high risk area (HRA); Regular = HRA breakdowns detected by regular herd tests; Recurrent = HRA breakdowns detected by post breakdown tests; Movement = HRA breakdowns detected by pre-movement herd tests; Slaughter = HRA breakdowns detected by slaughterhouse surveillance; OTFW = HRA OTFW breakdowns; LRA Cattle = Number of cattle infected while in unrestricted herds in the low risk area; LRA Breakdowns = Total breakdowns in LRA; LRA OTFW = OTFW breakdowns in LRA. All values except baselines are the averages of 10 simulations. Cases in which outputs appear to have low sensitivity to parameter values are shown in grey only to improve visual clarity.

may be considered a potential alternative to transmission from wildlife. However, persistent infection could only generate a limited proportion of observed breakdowns. The average number of OTFW breakdowns in the HRA in 2010 from the baseline model was 2153 (SD from 200 simulations was 58). Eliminating all persistent infection from the baseline model reduced this by less than 18% to 1778 (average of 10 simulations), while assuming that infection persisted whenever possible only increased the number of breakdowns by 26% to 2705. Moreover, the number of OTFW breakdowns in the LRA was more sensitive to variation of the persistence of infection: baseline 54 (SD 7), zero persistence 45, maximum persistence 88. Thus, a general increase of the persistence of infection would make it more difficult to match the observed contrast between the numbers of breakdowns in the HRA and LRA.

- 1. Sensitivity and specificity of surveillance tests

Increasing sensitivity of surveillance tests reduced numbers of cattle infected, but had a mixed effect on breakdowns. The number of breakdowns detected by post breakdown tests increased, while the number detected by slaughterhouse surveillance decreased. Increased sensitivity also reduced infected cattle and breakdowns in the LRA.

The specificity of the skin test most strongly influenced outputs that were relatively insensitive to other parameter values: i.e. the number of breakdowns in the LRA and the numbers of breakdowns detected by regular herd tests and pre-movement tests, which were surveillance streams with relatively low proportions of infected cattle and high proportions of OTFS breakdowns. Thus the number of breakdowns in the LRA was more sensitive to the specificity of the skin test than any other parameter. Hence the model outputs suggest that false positive skin tests generated significant numbers of OTFS breakdowns.

- 1. Confirmation and slaughterhouse surveillance

An apparent anomaly in the fitted baseline set of parameter values was the high sensitivity of surveillance at slaughter (maximum 0.875), which exceeded the probability that an infected reactor would be confirmed (maximum 0.40) (Table 2), although relatively recent estimates of slaughterhouse surveillance sensitivity have approached 0.7 (O'Hare et al., 2014). The probability that an infected reactor was confirmed was relatively easily estimated, because the only outputs sensitive to it were the numbers of OTFW breakdowns (Figure 4). Similarly, the only impact on outputs of the sensitivity of surveillance at slaughter was to increase the number of breakdowns detected by the surveillance at slaughter stream. A high sensitivity of surveillance at slaughter was required to match model outputs to the large number of breakdowns detected by surveillance at slaughter in 2010.

The model does not include approved finishing units (AFU) because they should not be a potential source of infection in other herds. However, because they keep cattle from herds under TB restrictions for finishing for a short time before slaughter, they provide a direct route to slaughterhouse surveillance with little exposure to other surveillance streams. Therefore the slaughterhouse cases from AFUs are additional to those that can be generated by the model. The current model also uses the same slaughterhouse surveillance sensitivity for all ages of cattle. In fact there is evidence suggesting that slaughterhouse surveillance may be most sensitive at close to the peak age for slaughter at about 2 years old (Brooks-Pollock et al., 2013).

1. **OUTPUTS**

The model generates up to three output files from each run of a simulation, as well as a single summary file collating all the runs of a simulation. User controlled flags in the file “configProperties.txt” set whether each simulation run outputs files with the final distribution of infected cattle, the final distribution of environmental infection states and a listing of breakdowns during the run.

- 1. Summary outputs

The user defines summary regions for each simulation so that a line of summary outputs for each summary region are added to a file named “modelSummary.csv” every year of every simulation run.

- - 1. Summary regions are defined as lists of counties, so that the highest resolution for summary outputs is to produce annual outputs for every county. Summary regions are defined using a simple table in the file “SummaryRegions.csv” to insert the name of each summary region against the counties it includes. Figure 5 shows the locations of counties by county ID and their management areas.
    2. The summary outputs produced for every summary region every year from every run of a simulation are:
- SimID: The ID of the simulation run (indexed as successive integers from the initial ID for each simulation).
- Year: The year, starting from 1 for the first year of the simulation.
- DaysInYear: The number of days in the year (allows identification of leap years).
- Region: The summary region.
- UndetectedHoldings: The number of holdings in the simulation that contain undetected infected animals at the end of the year.
- InOverflow: This is a diagnostic output to check the management of overflows of age bins in the model. (The static definition of holding size and animal age distribution can result in infected animals exceeding the capacity of holdings. Such animals are identified to prevent their loss and accelerate their removal to slaughter or other holdings.)
- Scotland_Mvmts: The number of infected animals that have been missed by pre-movement testing and subsequently moved to a holding located in Scotland.
- Slaughter_Mvmts: The number of infected animals that have been missed by slaughterhouse testing and subsequently slaughtered.
- Bin_Full: Another diagnostic output relating to age bins like “InOverflow”.
- Inf_From_Cattle: The number of new *M. bovis* cattle infections caused by cattle to cattle transmission.
- Inf_From_Wildlife: The number of new *M. bovis* cattle infections caused by transmission from environmental reservoirs.
- Slaughter_Test_Breakdowns: The number of holdings that have entered breakdowns due to failing a slaughterhouse test.
- Movement_Test_Breakdowns: The number of holdings that have entered breakdowns due to failing a pre-movement test.
- Interval_Test_Breakdowns: The number of holdings that have entered breakdowns due to failing a Generic herd test.
- Regular_Test_Breakdowns: The number of holdings that have entered breakdowns due to failing a regular herd test (annual, four-yearly, etc) test.
- Postbreakdown_Test_Breakdowns: The number of holdings that have entered breakdowns due to failing a post-breakdown test; the sum of:
- Failed 6-month post-breakdown tests.
- Failed 12-month post-breakdown tests (18 months after the end of a breakdown).
- Breakdowns: The total number of breakdowns, which should equal the sum of the 5 previous outputs.
- Confirmed_Breakdowns: A subset of Breakdowns including only those breakdowns which had at least one animal confirmed positive by detecting a lesion or bacterial culture.
- False_Positive_Breakdowns: The number of breakdowns which were initiated by a false-positive test result.
- Reactors: The total number of infected animals that were detected on holdings that entered breakdowns (not including false-positives).
- Confirmed_Reactors: A subset of Reactors including only those animals that had infection confirmed by detecting a lesion or bacterial culture.
- False_Positive_Reactors: The total number of animals which have given a false-positive reaction to a test for bTB, either in a disclosing test or during a breakdown.
- Generic_Tests: The total number of animal tests performed in generic herd tests.
- Regular_Tests: The total number of animal tests performed in regular herd tests.
- Post_Breakdown_Tests: The total number of animal tests performed in post breakdown tests.
- Pre_Movement_Tests: The total number of pre-movement tests performed.
- Slaughter_Tests: The total number of animals going to slaughter.
- Within_Breakdown_Tests: The number of short interval tests performed during breakdowns, estimated from the length of each breakdown and the herd size.

Figure 5: Regions indicated by the two digit CPH county IDs used for defining regions in summary outputs, with the 4 bTB management areas indicated by county used for model fitting. Note that the actual boundary between the High Risk Area and Edge was within counties during 2013-2017, and Derbyshire (09) is now in the Edge area from 1/01/2018.

- 1. Breakdown outputs

If “output.breakdownsSummary = true” in the file “configProperties.txt”, every run of a simulation generates a file “outputBreakdowns*nnn*.csv”, where *nnn* is the index number of the run, which lists all breakdowns during the run. Each breakdown provides a single record (row) including:

- Index: An ID number for the breakdown.
- CPH: The CPH ID of the breakdown holding.
- Month_start: The simulation month in which the breakdown started (first month recorded as 0).
- Day_start: The simulation day on which the breakdown started (first day recorded as 0).
- Month_end: The month when the breakdown ended (the first day of the month).
- SIT: The number of short interval testing times in the breakdown, which defines the breakdown duration.
- Type: Identifies the surveillance stream that initiated the breakdown.
- Status: Identifies whether the breakdown ended as OTFW (equivalent to confirmed) or OTFS (unconfirmed).
- Infected: Identifies whether the breakdown included an infected reactor.
- Region: The user-defined summary region including the breakdown holding.
- Persistent: Records whether an infected animal remained at the holding at the end of the breakdown.
- Num_cases: The total number of cases in the breakdown from the disclosing test and short interval tests, including infected and uninfected reactors and slaughterhouse cases.
- Infected _reactors: The number of reactors in the breakdown that were infected.
- Visible_lesions: The number of infected reactors that had visible lesions or bacterial culture.
- Slaughter_cases: The number of cases detected at slaughter during the breakdown.
- False_positive: The number of reactors in the breakdown that were not infected.
  1. Final distribution of infected cattle

If “output.animals = true” in the file “configProperties.txt”, every run of a simulation generates a file “infectedAnimals*nnn*.csv”, where *nnn* is the index number of the run, which lists all infected cattle remaining at the end of the run. Every cow is listed as a single record, including:

- CPH: The CPH ID of the breakdown holding.
- Age: The age in days of the infected animal.
- Days_infected: The number of days for which the animal has been infected.
  1. Final infection state of environmental reservoirs

If “output.wildlifeStates = true” in the file “configProperties.txt”, every run of a simulation generates a file “wildlifeState*nnn*.csv”, where *nnn* is the index number of the run, which lists all holdings in the simulation. Every holding is listed as a single record including:

- CPH: The CPH ID of the breakdown holding.
- Wildlife_state: An integer value 0, 1, 2 or 3 indicating the infection state of the holding’s environmental reservoir.

1. **ANONYMIZED MODEL**

A version of the model setup has been produced to demonstrate the model in the public domain.

- 1. Anonymization

All CPH identifiers in setup files have been altered to retain their county identifier, while replacing the parish and holding identifiers with values that uniquely identify each holding and maintain links to movement data, but are otherwise meaningless. All Northing and Easting locations have been changed to ‘111111’, and all holding types have been changed to ‘Beef’.

- 1. Validation

Ten replicates of confirmed breakdowns at county resolution generated from the compact, anonymized setup with baseline parameter settings in 2010 and 2016 were compared with the same outputs from the full setup with the same parameter settings. For each county the difference between the two setups was calculated as:

$$\frac{\chi^{2}}{\sqrt{10}}\approx\frac{\left( N_{anonymized}-N_{full} \right)^{2}}{\left( N_{full}+0.1 \right)}$$

The addition of 0.1 to the denominator was a compromise between avoiding division by zero and including regions that had a chance of no confirmed breakdowns in a year. The sum of these differences across 56 regions that had at least one confirmed breakdown in one of the 10 replicates of either of the setups was 12.2 in 2010 and 11.2 in 2016, which was consistent with the value of *χ*^2^_56_ that would be expected from the calculation, if the observations were randomly generated count data with the same set of expected values (95% confidence interval for *χ*^2^_56_/√10 ≈ 11.8 – 24.8).

1. **REFERENCES**

2014. The Strategy for achieving Officially Bovine Tuberculosis Free status for England. Department for Environment, Food and Rural Affairs, 85.

Álvarez, J., Bezos, J., de la Cruz, M.L., Casal, C., Romero, B., Domínguez, L., de Juan, L., Pérez, A., 2014. Bovine tuberculosis: Within-herd transmission models to support and direct the decision-making process. Res. Vet. Sci. 97, S61-S68.

Animal Health and Veterinary Laboratories Agency, 2010. SB4500: Bovine tuberculosis: Infection status in cattle annual surveillance report for the period January to December 2010. Defra, London, UK, 118.

Birch, C.P.D., 2006. Diagonal and orthogonal neighbours in grid-based simulations: Buffon's stick after 200 years. Ecol. Model. 192, 637-644.

Brooks-Pollock, E., Conlan, A.J.K., Mitchell, A.P., Blackwell, R., McKinley, T.J., Wood, J.L., 2013. Age-dependent patterns of bovine tuberculosis in cattle. Vet. Res. 44, 97.

Brooks-Pollock, E., Roberts, G.O., Keeling, M.J., 2014. A dynamic model of bovine tuberculosis spread and control in Great Britain. Nature 511, 228-231.

Broughan, J.M., Brouwer, A., Upton, P., 2014. Analysis of bovine tuberculosis surveillance at routine slaughter of cattle in Great Britain (2009-2013). AHVLA, Gov.uk, 80.

Conlan, A.J.K., McKinley, T.J., Karolemeas, K., Pollock, E.B., Goodchild, A.V., Mitchell, A.P., Birch, C.P.D., Clifton-Hadley, R.S., Wood, J.L.N., 2012. Estimating the Hidden Burden of Bovine Tuberculosis in Great Britain. PLOS Comput. Biol. 8, e1002730.

Delahay, R.J., Langton, S., Smith, G.C., Clifton-Hadley, R.S., Cheeseman, C.L., 2000. The spatio-temporal distribution of Mycobacterium bovis (bovine tuberculosis) infection in a high-density badger population. J. Anim. Ecol. 69, 428-441.

Downs, S.H., Clifton-Hadley, R.S., Upton, P.A., Milne, I.C., Ely, E.R., Gopal, R., Goodchild, A.V., Sayers, A.R., 2013. Tuberculin manufacturing source and breakdown incidence rate of bovine tuberculosis in British cattle, 2005-2009. Vet. Rec. 172, 98-+.

Dube, C., Stevenson, M.A., Garner, M.G., Sanson, R., Harvey, N., Estrada, C., Corso, B.A., Wilesmith, J.W., Griffin, J., 2007. Foot and mouth disease model verification and validation through a formal model comparison. Society for Veterinary Epidemiology and Preventive Medicine. Proceedings of a meeting held at Dipoli, Helsinki/Espoo, Finland, 28-30 March 2007, 124-140.

Goodchild, A.V., Downs, S.H., Upton, P., Wood, J.L.N., de la Rua-Domenech, R., 2015. Specificity of the comparative skin test for bovine tuberculosis in Great Britain. Vet. Rec. 177, 258.

Mitchell, A., Bourn, D., Mawdsley, J., Wint, W., Clifton-Hadley, R., Gilbert, M., 2005. Characteristics of cattle movements in Britain - an analysis of records from the Cattle Tracing System. Animal Science 80, 265-273.

Nuñez-Garcia, J., Downs, S.H., Parry, J.E., Abernethy, D.A., Broughan, J.M., Cameron, A.R., Cook, A.J., de la Rua-Domenech, R., Goodchild, A.V., Gunn, J., More, S.J., Rhodes, S., Rolfe, S., Sharp, M., Upton, P.A., Vordermeier, H.M., Watson, E., Welsh, M., Whelan, A.O., Woolliams, J.A., Clifton-Hadley, R.S., Greiner, M., 2017. Meta-analyses of the sensitivity and specificity of ante-mortem and post-mortem diagnostic tests for bovine tuberculosis in the UK and Ireland. Prev. Vet. Med.

O'Hare, A., Orton, R.J., Bessell, P.R., Kao, R.R., 2014. Estimating epidemiological parameters for bovine tuberculosis in British cattle using a Bayesian partial-likelihood approach. Proc. R. Soc. B 281.

Smith, G.C., Delahay, R.J., McDonald, R.A., Budgey, R., 2016. Model of selective and non-selective management of badgers (Meles meles) to control bovine tuberculosis in badgers and cattle. PLOS One 11.

Thom, M.L., Hope, J.C., McAulay, M., Villarreal-Ramos, B., Coffey, T.J., Stephens, S., Vordermeier, H.M., Howard, C.J., 2006. The effect of tuberculin testing on the development of cell-mediated immune responses during mycobacterium bovis infection. Vet. Immunol. Immunopathol. 114, 25-36.
